# No evidence of quantitative signal honesty across species of aposematic burnet moths (Lepidoptera: Zygaenidae)

**DOI:** 10.1111/jeb.13389

**Published:** 2018-11-02

**Authors:** Emmanuelle S. Briolat, Mika Zagrobelny, Carl E. Olsen, Jonathan D. Blount, Martin Stevens

**Affiliations:** ^1^ Centre for Ecology & Conservation College of Life & Environmental Sciences University of Exeter Penryn UK; ^2^ Plant Biochemistry Laboratory and Copenhagen Plant Science Centre Department of Plant and Environmental Sciences University of Copenhagen Copenhagen Denmark

**Keywords:** aposematism, comparative studies, cyanogenic glucosides, defence, insects, signal honesty, *Zygaena*

## Abstract

Many defended species use conspicuous visual warning signals to deter potential predators from attacking. Traditional theory holds that these signals should converge on similar forms, yet variation in visual traits and the levels of defensive chemicals is common, both within and between species. It is currently unclear how the strength of signals and potency of defences might be related: conflicting theories suggest that aposematic signals should be quantitatively honest, or, in contrast, that investment in one component should be prioritized over the other, while empirical tests have yielded contrasting results. Here, we advance this debate by examining the relationship between defensive chemicals and signal properties in a family of aposematic Lepidoptera, accounting for phylogenetic relationships and quantifying coloration from the perspective of relevant predators. We test for correlations between toxin levels and measures of wing colour across 14 species of day‐flying burnet and forester moths (Lepidoptera: Zygaenidae), protected by highly aversive cyanogenic glucosides, and find no clear evidence of quantitative signal honesty. Significant relationships between toxin levels and coloration vary between sexes and sampling years, and several trends run contrary to expectations for signal honesty. Although toxin concentration is positively correlated with increasing luminance contrast in forewing pattern in 1 year, higher toxin levels are also associated with paler and less chromatically salient markings, at least in females, in another year. Our study also serves to highlight important factors, including sex‐specific trends and seasonal variation, that should be accounted for in future work on signal honesty in aposematic species.

## 1 INTRODUCTION

Aposematic animals use conspicuous colours and patterns to warn potential predators of their unprofitability, linked to physical or chemical defences (Ruxton, Sherratt, & Speed, 2004; Stevens & Ruxton, 2012). This strategy, first proposed by Alfred Russell Wallace to explain the colourful appearance of caterpillars (Wallace, 1867), is now recognized to occur in a wide range of taxa, from a host of invertebrates [e.g. Hemiptera (Exnerová et al., 2003), Lepidoptera (Rothschild, 1985)] and amphibians (e.g. poison frogs; Summers & Clough, 2001) to mammals (Stankowich, Caro, & Cox, 2011) and birds (Dumbacher, Deiner, Thompson, & Fleischer, 2008). Predators who encounter distasteful warningly coloured prey should learn to associate the prey signal with their unpleasant experience and avoid attacking similar prey in the future. Bright and colourful patterns facilitate this process in a number of ways, enhancing the “efficacy” of aposematic signals by increasing their detectability, memorability and discriminability (Guilford & Dawkins, 1991; Ruxton et al., 2004). Moreover, traditional theory rooted in Fritz Müller's insights into mutually beneficial mimicry between defended species (Müller, 1879) holds that warning signals should converge onto a limited number of common forms, to further speed up predator avoidance learning. Yet, there is extensive variation in warning coloration across aposematic taxa, which can be perceptible to their predators (Arenas & Stevens, 2017; Briolat, Burdfield‐Steel, et al., 2018).

A key line of enquiry into this seemingly paradoxical variation explores the relationship between the strength of visual signals and levels of defences, which also vary greatly both between (e.g. Arenas, Walter, & Stevens, 2015) and within species (e.g. Brower, Ryerson, Coppinger, & Glazier, 1968). As conspicuous coloration incurs the cost of heightened detection by predators, it should often be too costly for undefended species, which would be captured and consumed (with the exception of Batesian mimics of aposematic species; Bates, 1862). Aposematic signals are therefore generally considered to be qualitatively honest, reliably indicating the presence of a defence (Ruxton et al., 2004; Sherratt, 2002). Whether they should also be expected to be quantitatively honest, with the strength of visual signals reflecting the potency of the defences they advertise, is more controversial.

Despite the cost of increased visibility to predators, early interpretations of aposematism as an honest handicap signal (Grafen, 1990) have been criticized for the lack of a physiological link between visual features and defensive chemistry (Guilford & Dawkins, 1993). This could be provided by competition between traits for resources, leading to positive correlations between signals and defences when these resources are limited (Blount, Speed, Ruxton, & Stephens, 2009; Blount et al., 2012). Yet, some theoretical models predict a disjunction between signals and defences, suggesting that prey should prioritize investment in either signals, to which predators respond (Leimar, Enquist, & Sillen‐Tullberg, 1986), or defences, which do not incur detection costs (Speed & Ruxton, 2007). Overall, considering the relative costs of signals and defences, quantitative honesty may be expected to occur under certain conditions, depending on the economics of colour and toxin production (Speed & Ruxton, 2007), predator behaviour (Guilford, 1994; Speed, Ruxton, Blount, & Stephens, 2010) and prey resilience to attack (Sherratt, 2002). Although most theoretical work focuses on single species, several of these evolutionary mechanisms have been proposed to underpin signal honesty across closely related species too (Summers, Speed, Blount, & Stuckert, 2015). Coevolutionary dynamics with mimics of defended prey (Franks, Ruxton, & Sherratt, 2009), cautious or “go‐slow” behaviour on the part of predators (Guilford, 1994), exaptation through other functions of visual signals (Lee, Speed, & Stephens, 2011), and resource allocation trade‐offs (Blount et al., 2009), are all thought to have the capacity to lead to honest signalling between populations or species (Holen & Svennungsen, 2012; Summers et al., 2015).

Most empirical studies of the relationship between signals and defences across clades of species have found positive correlations between measures of visual signal strength and measures of toxicity, suggesting quantitative honesty in signalling (Cortesi & Cheney, 2010; Santos & Cannatella, 2011; Summers & Clough, 2001; but see Darst, Cummings, & Cannatella, 2006; Winters et al., 2018). Work on ladybird beetles (Coccinellidae), combining toxin bioassays and field predation experiments with ladybird models presented to birds, has explicitly linked more conspicuous coloration and higher defence levels to greater survival in the wild (Arenas et al., 2015). However, these studies are restricted in taxonomic scope, primarily focusing on poison frogs (Dendrobatidae), ladybird beetles and to a lesser extent marine opisthobranchs (Cortesi & Cheney, 2010; Winters et al., 2018), so research in a wider range of taxa is needed before more general conclusions can be drawn (Stevens, 2015; Summers et al., 2015). Existing studies can also be difficult to compare, as they employ a wide range of methods for quantifying defences, from bioassays (e.g. Arenas et al., 2015; Darst et al., 2006) to specific quantification of individual chemicals (e.g. alkaloids in the Dendrobatidae; Summers & Clough, 2001), and vary in their approaches to measuring coloration. Animal visual systems differ from human perception and are highly variable between species, so it is essential to consider visual signals from the perspective of the relevant receivers, which in the case of aposematism are potential predators (Stevens, 2007, 2011). Although this is not always the case (e.g. Dumbacher, Spande, & Daly, 2000; Dumbacher et al., 2008; Summers & Clough, 2001), studies of aposematism are increasingly considering predator perception [e.g. birds (Arenas et al., 2015; Darst et al., 2006) and fish (Cortesi & Cheney, 2010; Winters et al., 2018)], as our understanding of animal vision improves.

Aposematic burnet moths (Lepidoptera: Zygaenidae) are well suited to testing the relationship between signals and defences across closely related species. In the Western Palearctic, the Zygaenidae are represented by three subfamilies: the Zygaeninae, Procridinae and Chalcosiinae. Of the 1036 species of Zygaenidae recognized worldwide (van Nieukerken et al., 2011), all 45 tested so far, including members of all three relevant subfamilies (38 Zygaeninae, including 35 *Zygaena* spp., two Procridinae and five Chalcosiinae), possess potent chemical defences, in the form of cyanogenic glucosides (Davis & Nahrstedt, 1982, 1985; Zagrobelny et al., 2004). The Zygaenidae synthesize the cyanogenic glucosides linamarin and lotaustralin de novo, from the amino acids valine and isoleucine, respectively (Wray, Davis, & Nahrstedt, 1983), but species in the Zygaeninae further have the apparently unique ability to simultaneously sequester the same compounds from their host plants (Zagrobelny et al., 2014). Cyanogenic glucosides, occurring in plants and several arthropod lineages (Zagrobelny, Bak, & Møller, 2008), are bitter‐tasting compounds, distasteful to avian predators, so are likely to facilitate taste rejection during an attack (Skelhorn & Rowe, 2009). They are also toxic, releasing hydrogen cyanide upon enzymatic breakdown, due to enzymes either in the gut of predators or present in the prey themselves (Zagrobelny et al., 2008). In terms of coloration, there are dramatic differences in wing patterns between subfamilies of Zygaenidae, and more subtle variation within. Burnet moths in the genus *Zygaena* are characterized by classically conspicuous aposematic markings, with a typical pattern of black forewings with red spots, and red hindwings. Both within and between species, there can be extensive variation on this theme, with respect to the colour, size, shape and number of markings (Hofmann & Tremewan, 2017). By contrast, temperate species of Procridinae, or forester moths, are iridescent green or dull brown in colour (Drouet, 2016) and are generally considered cryptic (Efetov & Tarmann, 1999). The single representative of the Chalcosiinae in Western Europe, *Aglaope infausta* (L.), has brown forewings with discreet red markings, and red hindwings.

To test for evidence of quantitative signal honesty across the Zygaenidae, we measured signal and defence properties in 14 species, collected in 2015 and 2016 from a range of locations in Denmark, France and the UK. As the defences of the Zygaenidae have been extensively studied, we were able to accurately quantify the levels of cyanogenic glucosides in our samples, using a liquid chromatography–mass spectrometry (LC‐MS) protocol specifically refined to identify linamarin and lotaustralin. In terms of signal receivers, birds are the most likely visually driven predators of adult Zygaenidae. Experiments with captive birds, including *Cyanistes caeruleus* (blue tits) and *Parus major* (great tits) (Wiklund & Järvi, 1982) as well as *Sturnus vulgaris* (starlings; Rammert, 1992), suggest that they generally find burnet moths distasteful, yet observations in the wild reveal that several species, such as *Alauda arvensis* (skylarks), *Anthus pratensis* (meadow pipits) and even *S. vulgaris,* will nevertheless attack and in some cases partly or entirely consume these moths (Tremewan, 2006). Using visual modelling techniques, we measured multiple characteristics of zygaenid wing patterns, from the perspective of a potential avian predator, with a visual system modelled on the blue tit, *C. caeruleus*. In addition, molecular data and recent phylogenies of the Zygaenidae and the genus *Zygaena* are available (Niehuis, Hofmann, Naumann, & Misof, 2007; Niehuis, Naumann, & Misof, 2006a,b,c), enabling evolutionary relationships to be accounted for when analysing variation across species. This study is the first detailed exploration of the chemical defences and coloration of multiple species in this family of aposematic Lepidoptera. We test the idea of quantitative signal honesty in a new study system, using relevant and meaningful measures of signals and defences, to contribute to the debate over signal honesty across aposematic species.

## 2 MATERIALS AND METHODS

### 2.1 Specimen collection and rearing

Individuals of 14 Zygaenidae species were collected in spring and summer 2015 and 2016, from locations in Denmark, France and the UK (Table 1; see Supporting Information Data S1 for full details). Where possible, host plants were sampled at the same locations (see Supporting Information Data S2). To ensure that all Zygaenidae analysed were virgin, an important consideration as males and females exchange cyanogenic glucosides during reproduction (Zagrobelny, Bak, Ekstrøm, Olsen, & Møller, 2007; Zagrobelny, Bak, Olsen, & Møller, 2007; Zagrobelny, Motawia, Olsen, Bak, & Møller, 2013), specimens were collected at the larval or pupal stage, then reared to maturity in the laboratory. Larvae and pupae were kept in individual boxes with air holes, inside an incubator at 20°C, with a 16:8 hr day:night cycle, following protocols from previous work on *Zygaena filipendulae* (Linnaeus, 1758) (Zagrobelny et al., 2007). The larvae were fed ad libitum with the same host plant as they were found on in the field (Table 1). After emergence, the adults were euthanized by placing them in a −80°C freezer. Due to the difficulty of finding larvae or pupae of certain species, and high mortality, five species are limited to very small sample sizes (*N* = 1 or *N* = 2, see Table 1). Their wings were dissected for photography, and then, the entire sample was placed in 1 ml 80% methanol in preparation for LC‐MS analysis of cyanogenic glucoside content.

**Table 1:** Number (*N*), species and host plants of photographed specimens

Species	Country	Host plant at collection site	*N*	
			2015	2016
*Aglaope infausta* (Linnaeus, 1767)	France	*Cotoneaster* sp., *Crateagus* sp., *Prunus* sp. (Rosaceae)	21	17
*Rhagades pruni* (Denis & Schiffermüller, 1775)	France	*Prunus spinosa* (Rosaceae)	8	8
*Theresimima ampellophaga* (Bayle‐Barelle, 1808)	France	*Vitis* sp. (Vitaceae)	0	1
*Zygaena cynarae* (Esper, 1789)	France	*Peucedanum cervaria* (Apiaceae)	1	0
*Zygaena ephialtes* (Linnaeus, 1767)	France	*Securigera varia* (Fabaceae)	21	0
*Zygaena erythrus* (Hübner, 1806)	France	*Eryngium campestre* (Apiaceae)	0	11
*Zygaena exulans* (Hohenwarth, 1792)^a^	France	Polyphagous – host plant unknown	0	5
*Zygaena filipendulae* (Linnaeus, 1758)	Denmark, France, UK	*Lotus corniculatus, Dorycnium pentaphyllum, Hippocrepis comosa* (Fabaceae)	107	8
*Zygaena lonicerae* (Scheven, 1777)	France	*Trifolium* sp. (Fabaceae)	0	1
*Zygaena minos* (Denis & Schiffermüller, 1775)	France	*Pimpinella saxifraga* (Apiaceae)	1	1
*Zygaena occitanica* (Villiers, 1789)	France	*Dorycnium pentaphyllum* (Fabaceae)	0	2
*Zygaena sarpedon* (Hübner, 1790)	France	*Eryngium campestre* (Apiaceae)	6	2
*Zygaena transalpina* (Esper, 1780)	France	*Hippocrepis comosa*,* Securigera varia* (Fabaceae)	3	13
*Zygaena trifolii* (Esper, 1783)	UK	*Lotus pedunculatus* (Fabaceae)	9	14

a Collected as pupae only.

### 2.2 Wing photography

Photographs of the moths’ forewings were taken with a calibrated, UV‐sensitive digital camera [Nikon D7000 (Nikon, Tokyo, Japan) fitted with a 105 mm CoastalOptics quartz lens], in controlled conditions inside a dark room. Lighting was provided by an EYE Color Arc Lamp MT70 bulb (Iwasaki Electric Co. Ltd., Tokyo, Japan), its UV‐blocking coating removed by lightly scrubbing with a steel brush (Troscianko & Stevens, 2015), thus emitting a spectrum of light similar to D65 daylight conditions. The forewings were chosen for analysis as they are more visible to predators than the hindwings, which in the Zygaenidae are hidden from view when at rest. As these wings are iridescent, only the right‐hand wings were photographed (to keep scale direction consistent), and the light source and camera were held at constant angles relative to the wings (50° and 90°, respectively). The wings were photographed flat against a background of grey ethylene‐vinyl acetate (EVA, or craft foam). A scale bar and a set of two polytetrafluoroethylene (PTFE) reflectance standards, reflecting 7% and 93% of all wavelengths of light, respectively (Zenith Lite Diffuse Target sheets, SphereOptics, Pro‐Lite Technology, Cranfield, UK), were included in each photograph, enabling calibration of the images with respect to lighting conditions (Troscianko & Stevens, 2015). Each specimen was photographed twice, using different filters [a UV/infrared blocking filter (Baader UV/IR Cut Filter), transmitting between 400 and 700 nm, and a UV pass and IR blocking filter (Baader U filter), transmitting between 300 and 400 nm). All photographs were taken in RAW format, with a constant aperture (f8) and manual white balance set to “cloudy”.

### 2.3 Image analysis

All image analysis was performed in ImageJ (Schneider, Rasband, & Eliceiri, 2012) using open access custom‐made plugins in the Image Calibration and Analysis Toolbox (Troscianko & Stevens, 2015). Methods used for processing images and extracting colour metrics are summarized below; full details are provided in Supporting Information Data S3. To allow for objective colour measurements, images were linearized and normalized (Stevens, Párraga, Cuthill, Partridge, & Troscianko, 2007), and then scaled to 100 pixels/mm. Photographs taken with the two types of filter were combined using an automatic alignment tool, and the resulting multispectral images were mapped to avian vision, as previous observations show that birds are likely to be the most relevant visual predators of burnet moths (Tremewan, 2006). Each image was converted to the visual system of *C. caeruleus*, the model species for the ultraviolet‐sensitive (UVS) avian visual system (Hart, Partridge, Cuthill, & Bennett, 2000) using a highly accurate polynomial mapping technique (Pike, 2011; Stevens & Cuthill, 2006; Stevens et al., 2007; Troscianko & Stevens, 2015) to produce a set of image layers corresponding to the predicted cone catch values for each of the five avian cone types: long wavelength (LW)‐, medium wavelength (MW)‐, short wavelength (SW)‐ and ultraviolet (UV)‐sensitive photoreceptors, and double cones. Relevant wing areas were selected using the freehand tool in ImageJ. Most species display red forewing markings, but for *Rhagades pruni* (Denis & Schiffermüller, 1775), the iridescent blue patch at the base of the wing was selected as the markings, whereas for *Theresimima ampellophaga* (Bayle‐Barelle, 1808), the whole uniform wing was measured as a single patch. Cone catch values for every photoreceptor type were obtained from each selected patch and then averaged to obtain a single measure of colour per individual, for both the wing markings and wing background area.

### 2.4 Colour metrics

Based on the average cone catch values, several measures of coloration were calculated: luminance, saturation and hue of the forewing marking colours, as well as both chromatic and luminance contrasts between markings and background colours. In brief, luminance (perceived lightness) was taken as the cone catch value for the double cones (Jones & Osorio, 2004; Osorio & Vorobyev, 2005), and saturation, measuring colour “richness”, was calculated by plotting wing colours in a tetrahedral colour space and measuring the Euclidian distance from each colour to the centre of the tetrahedron (after Endler & Mielke, 2005; Stoddard & Prum, 2008). Hue, representing the type or shade of a colour, was derived using principal component analysis (after Spottiswoode & Stevens, 2011) to obtain a ratio of cone catch values broadly inspired by the general principle of colour opponency, known to be relevant to avian vision (Osorio, Jones, & Vorobyev, 1999; Osorio, Vorobyev, & Jones, 1999). In this study, hue is given by the following equation, such that higher hue values represent colours with relatively higher reflectance in the LW or UV channels, indicating redder colours, higher ultraviolet reflectance or both:

(1) Hue=(LW+UV)/(SW+MW)

Chromatic and achromatic contrasts between the markings and background colours provide a sense of the salience of wing markings, and may be relevant to predator behaviour, although the relative importance of pattern contrast over colour *per se* in aposematic signals remains unclear (Aronsson & Gamberale‐Stille, 2008, 2012a,b; Svádová et al., 2009). Internal contrasts were calculated using a log version of the Vorobyev–Osorio model (Vorobyev & Osorio, 1998) and relative cone abundance values for *Cyanistes caeruleus* as a model for the UVS avian visual system (Hart et al., 2000), with a widely used estimate of the Weber fraction (ω* *= 0.05; Eaton, 2005; Håstad, Victorsson, & Ödeen, 2005; Stevens, 2011) to calculate noise. Achromatic, or luminance, contrast was taken as the natural logarithm of the ratio between the mean double cone catch values of two colours, divided by the same Weber fraction (Siddiqi, Cronin, Loew, Vorobyev, & Summers, 2004). Both contrasts are measured in “just‐noticeable differences” (JNDs): values below 1 suggest that the two colours compared are indiscriminable, even in optimal lighting conditions, whereas values above 1 and higher indicate colours increasingly easy to discriminate (Siddiqi et al., 2004). Supporting Information Data S3 provides details on the calculations of all the metrics described above.

### 2.5 Quantification of chemical defences

After photography, each specimen, complete with its forewings, was preserved in 1 ml 80% methanol in preparation for analysis of their cyanogenic glucoside content. Quantification of linamarin and lotaustralin in our samples was performed by liquid chromatography–mass spectrometry (LC‐MS), following a protocol specifically refined to identify these compounds, and used in previous work on the chemistry of the Zygaenidae (Fürstenberg‐Hägg et al., 2014; Pentzold et al., 2015, 2016; Zagrobelny, Simonsen, Olsen, Bak, & Møller, 2015; Zagrobelny et al., 2004, 2007, 2007, 2014). Samples were prepared by grinding up the specimens in 1 ml ice‐cold 55% MeOH with 0.1% formic acid and then passing them through an Anopore 0.45‐μm filter (Whatman). The analytical LC‐MS was performed with an Agilent 1100 Series LC (Agilent Technologies, Waldbronn, Germany), and Bruker HCT‐Ultra ion trap mass spectrometer (Bruker Daltonics, Bremen, Germany), run in positive electrospray mode, with an oven temperature of 35°C. A Zorbax SB‐C18 column (Agilent; 1.8 μM, 2.1 × 50 mm) was used for chromatographic separation, running with a flow rate of 0.2 ml/min, increased to 0.3 ml/min from 11.2 to 13.5 min. The mobile phases A and B were composed, respectively, of H_2_O with 0.1% (v/v) HCOOH, 50 μM NaCl and MeCN with 0.1% (v/v) HCOOH, with a gradient as follows: 0–0.5 min, isocratic 2% B; 0.5–7.5 min, linear gradient 2%–40% B; 7.5–8.5 min, linear gradient 40%–90% B; 8.5–11.5 isocratic 90% B; 11.6–17 min, isocratic 2% B. Sodium adducts of linamarin [retention time (RT) 2.6 min, (M + Na)^+^ at *m/z* 270] and lotaustralin [RT 5.5 min, (M + Na)^+^ at *m/z* 284] were detected and compared to authentic standards (Møller, Olsen, & Motawia, 2016) using native analysis software. The total amount of each compound was estimated according to its extracted ion chromatogram (EIC) peak areas and quantified using calibration curves for the linamarin, lotaustralin and amygdalin standards. Finally, the concentration of cyanogenic glucosides in each sample was determined by dividing the total amount of compounds in each sample by the specimen mass, recorded at the time of preservation. Samples of larval host plants were analysed similarly. To rule out the possibility that differences between samples from 2015 and 2016 were caused by the LC‐MS machine, a subset of 20 samples (5 *A. infausta* and 5 *Z. trifolii* from each year, both males and females) were run together a second time in 2017. Analysing the results with a mixed effects model including specimen ID as a random effect, we found no significant effect of the interaction between collection year and machine run (original, in 2015 or 2016, vs. second run in 2017) on the concentration of cyanogenic glucosides for either *A. infausta* ($\chi_{1}^{2}$ = 1.73, *df* = 1, *p* = 0.19) or *Z. trifolii* ($\chi_{1}^{2}$ = 0.64, *p *= 0.43), suggesting that differences between years were not due to variation in the sensitivity of the LC‐MS machine in 2015 and 2016.

### 2.6 Phylogenetic reconstruction

The phylogenetic tree was reconstructed using previously published mitochondrial and nuclear sequences, following existing studies of the evolutionary history of the Zygaenidae (Niehuis et al., 2006a, 2007): complete sequences of the mitochondrial genes for NADH dehydrogenase subunit 1 (ND1), tRNA‐leucine (tRNA‐Leu), the large subunit ribosomal RNA (16S rRNA), tRNA‐valine (tRNA‐Val) and a large fragment of the sequence for the mitochondrial small subunit of rRNA (12S rRNA), as well as two nuclear DNA fragments, an almost complete sequence of the small subunit rRNA (18S rRNA) and the 5′ end of the large subunit rRNA (28S rRNA). A new phylogenetic tree was built from these sequences, as previously published phylogenies using all available sequences (Niehuis et al., 2006a, 2007) did not include all our species of interest. *Sesia bembeciformis* (Lepidoptera: Sesiidae) was used as an outgroup to root the tree (Niehuis et al., 2006a,b,c). Sequences for each species photographed and the outgroup were downloaded from GenBank (http://www.ncbi.nlm.nih.gov/; see Supporting Information Data S4) and aligned using MUSCLE (Edgar, 2004), as implemented by the “ape” package (Paradis, Claude, & Strimmer, 2004) in R 3.3.1 (R Development Core Team, 2015). The alignments for each sequence were then concatenated to produce a single final alignment [5697 base pairs (bp) long] for phylogenetic reconstruction.

**Figure 1:**
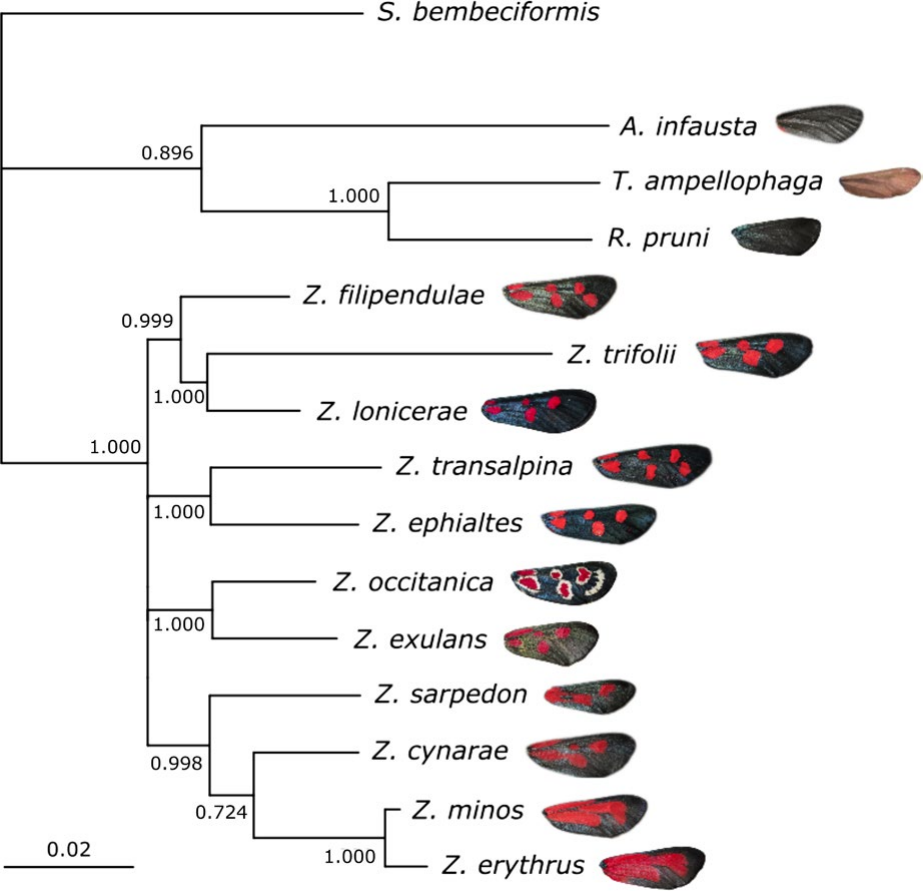
Phylogenetic tree of the Zygaenidae used in this study. Branch labels represent bootstrap values for 1000 replicates; the scale bar corresponds to genetic distances between sequences, along branch lengths. Image credits: *T. amphellophaga,* adapted from www.lepinet.fr/especes/nation/lep/index.php?id=02140, ©Daniel Morel; all other images authors’ own

Phylogenetic relationships were assessed with maximum likelihood (ML), using the “phangorn” package (Schliep, 2011) in R. The most appropriate model of evolution was identified as a GTR+G+I model, allowing for variation in mutation rates between sites and the presence of invariant sites, according to ML estimates calculated with the modelTest function in “phangorn”. Tree topology was then optimized by nearest‐neighbour interchange (NNI), using the optim.pml function. Finally, partitions allowing different rates of evolution for nuclear and mitochondrial sequences or for every different gene were tested with the pmlPart function. Based on Akaike information criterion (AIC) scores, a partitioned model considering each gene separately was selected (AIC_no partition_ = 40049.83, AIC_nuclear/mitochondrial partition_ = 39575.70, AIC_partition by gene_ = 39405.41). The final rooted tree (Fig. 1) was bootstrapped with 1,000 replicates, and nodes with <70% support were collapsed into polytomies.

### 2.7 Statistical analyses

All analyses were carried out in R 3.3.1 (R Development Core Team, 2015). To test whether data collected in 2015 and 2016 could be analysed together, we examined differences in cyanogenic glucoside concentration and colour metrics (luminance, saturation, hue, internal contrasts and relative marking area on the forewing) between years, across the seven species collected in both (see Table 1). These were tested for each dependent variable in turn, with a linear model allowing interactions between the independent variables of year, sex and species in the full model, followed by model simplification. Luminance, hue and chromatic contrast were log‐transformed to fit model assumptions.

As this investigation revealed significant effects of year and sex on both toxicity and colour metrics, we subsequently analysed the relationship between colour metrics and cyanogenic glucoside levels across species separately for each year. The data were also analysed across both sexes, and for males and females separately. To account for evolutionary relatedness between species, we used phylogenetic generalized least squares (PGLS) models, allowing *λ* to be fitted by maximum likelihood (Mundry, 2014), as implemented by the package “caper” (Orme, 2013). We set out to test the relationship between cyanogenic glucoside concentration and all available colour metrics in a single model, but several of these variables were highly correlated. To deal with the problem of collinearity, we calculated variance inflation factors (VIFs) using the vif function in the “car” package (Fox & Weisberg, 2011) and selected appropriate models by a combination of a commonly used “rule‐of‐thumb”, whereby VIFs should not exceed 10, and logical expectations of correlations (Dormann et al., 2013; O'Brien, 2007): for example, colour measures such as saturation, hue and chromatic contrast are calculated from the same cone catch values, so are expected to be correlated, whereas marking size is not tied to these variables. This yielded 3–4 different models per data set (combination of sex and collection year; see Supporting Information Data S5). To fit model assumptions, for the data set of females in 2015, saturation was transformed using the square‐root function, and chromatic contrast was log‐transformed. Cyanogenic glucoside concentration was log‐transformed for all the 2016 data sets. Finally, small phylogenies suffer from a lack of power (Freckleton, Harvey, & Pagel, 2002), making it difficult to accurately estimate parameters of phylogenetic signal, such as *λ* (Arenas et al., 2015; Symonds & Blomberg, 2014). We thus re‐ran the same PGLS models with *λ* fixed to 1, corresponding to a Brownian model of evolution, to check whether our results were affected by a low estimate of phylogenetic signal.

With the exception of *Zygaena filipendulae*, for which quantitative signal honesty has already been investigated (Briolat, Zagrobelny, Olsen, Blount, & Stevens, 2018), sample sizes in this study are generally too low to explore intraspecific variation in toxin level and coloration, especially as the different collection years and localities used for each species would also have to be accounted for (see Supporting Information Table S1). However, we do investigate quantitative honesty in *Z. ephialtes*, a species for which all samples (*N* = 21) originated from a single location in 2015 (see Supporting Information Data S6). Following Briolat, Zagrobelny, et al. (2018), we used multiple linear regression and stepwise model simplification to test the relationship between the concentration of cyanogenic glucosides in each sample and forewing coloration. As above, VIFs were used to determine that models including saturation or hue should be run separately. Models included all other possible colour metrics (luminance, chromatic contrast, luminance contrast, relative marking area, and either hue or saturation), and sex was allowed to interact with every metric.

## 3 RESULTS

### 3.1 Within species, signals and defences vary between years and between sexes

**Figure 2:**
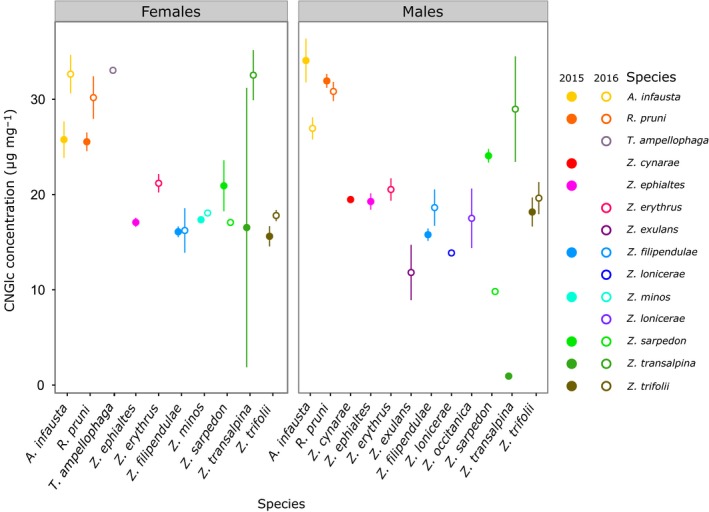
Mean and standard error of the concentration of cyanogenic glucosides (CNGlc) in males and females of each species. Filled circles = samples collected in 2015; open circles = samples collected in 2016

Analysing data from the seven species collected in both 2015 and 2016 revealed significant interactions between sex, year and species when testing for differences in both cyanogenic glucoside concentration and measures of colour (Table 2). Differences in cyanogenic glucoside concentration between years varied across species and between sexes. Cyanogenic glucoside levels in females increased between 2015 and 2016 in most species, with the exception of *Z. sarpedon*; in males, a more complex picture emerged, with half the species showing an increase in toxicity between years, and half showing a decrease (Figure 2).

**Table 2:** Results of stepwise simplification of models testing differences in cyanogenic glucoside (CNGlc) concentration and colour metrics between 2015 and 2016

Metric	Factor	*F*	*df*	*p*	Significance
CNGlc concentration	Sex:Species:Year	3.21	5, 192	0.0083	**
Luminance	Sex:Species:Year	2.35	5, 192	0.042	*
Saturation	Sex:Species:Year	1.42	5, 192	0.22	–
	Sex:Year	0.17	1, 197	0.68	–
	Sex:Species	1.49	5, 198	0.20	–
	Species:Year	4.17	6, 203	<0.001	***
	Sex	5.87	1, 203	0.016	*
Hue	Sex:Species:Year	0.82	5, 192	0.54	–
	Sex:Year	0.061	1, 197	0.80	–
	Sex:Species	1.53	5, 198	0.18	–
	Species:Year	27.95	6, 203	<0.001	***
	Sex	4.99	1, 203	0.027	*
Chromatic contrast (JNDs)	Sex:Species:Year	0.47	5, 192	0.80	–
	Sex:Year	0.0056	1, 197	0.94	–
	Sex:Species	3.08	5, 198	0.011	*
	Species:Year	3.32	6, 198	0.0039	**
Achromatic contrast (JNDs)	Sex:Species:Year	1.12	5, 192	0.35	–
	Sex:Year	2.06	1, 197	0.15	–
	Sex:Species	5.57	5, 198	<0.001	***
	Species:Year	10.67	6, 198	<0.001	***
Relative marking area (%)	Sex:Species:Year	0.84	5, 192	0.35	–
	Sex:Year	0.0013	1, 197	0.97	–
	Sex:Species	5.45	5, 198	<0.001	***
	Species:Year	2.97	6, 198	0.0085	**

Significance levels: *p < 0.05, **p < 0.01, ***p < 0.001.

**Figure 3:**
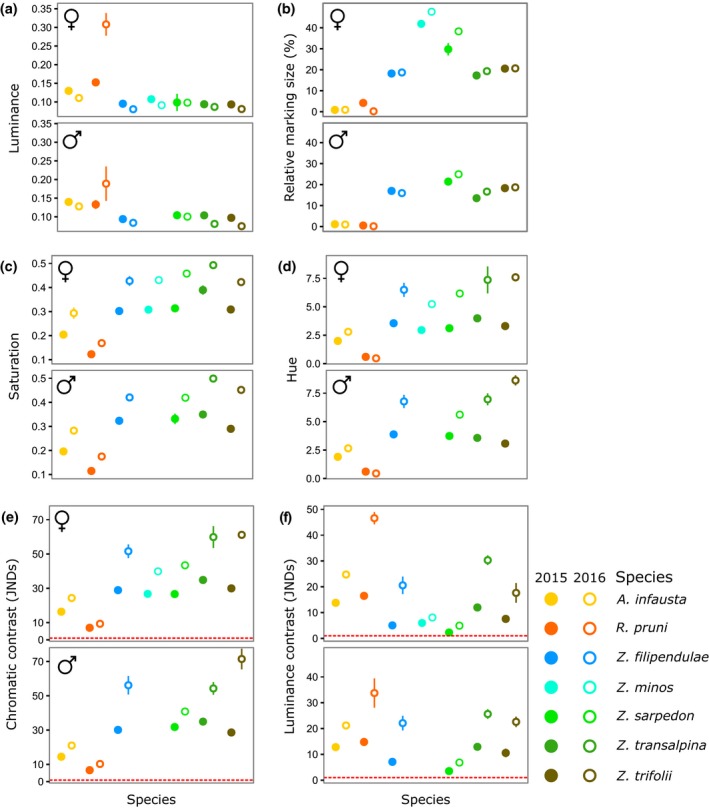
Mean values and standard errors of coloration for males and females of species collected in 2015 and 2016. Filled circles = samples collected in 2015; open circles = samples collected in 2016. In (b), relative marking size is measured as the percentage of the forewing area occupied by contrasting markings. In (e) and (f), the red dashed line represents the threshold for discrimination, JND = 1

With regard to coloration, there was a significant interaction between year and species for all colour metrics analysed (Table 2). Individuals of all species collected in 2016 consistently displayed features suggesting that their markings would be more salient to predators (Figure 3). Specimens of species with red wing markings collected in 2015 had paler wing markings than those found in 2016, although the extent of the difference varied between species and sexes (Figure 3a; Table 2). They also displayed markings with higher saturation and hue values, more contrasting against the wing background colours, and occupying a larger proportion of the forewing (Figure 3b–f). This indicates that their markings had more intense colours, which were also relatively redder (or had higher UV reflectance), larger and more conspicuous. For *Rhagades pruni*, which displays iridescent blue markings, trends in luminance and hue were opposite to those seen in all other species (Figure 3a,d). Nevertheless, this led to similar effects on marking saturation and internal contrasts in the forewings, which were also higher in 2016 than 2015 in this species (Figure 3c,e). Differences in the levels of signals and defences between years cannot be fully elucidated with samples from only 2 years but may be linked to variation in climate and environmental conditions (see Supporting Information Data S7). As sex and year influenced both colour metrics and cyanogenic glucoside levels, these variables could not be ignored in cross‐species analyses of signal honesty. Subsequent tests of the relationship between colour and toxicity were thus carried out separately for each year and each sex.

In *Z. ephialtes*, for which sufficient samples were collected in a single year and location, some significant associations were found between cyanogenic glucoside levels and measures of coloration. Toxin levels increased with relative marking size in males, but decreased in females (linear model, *F*_1,16_ = 23.50, *p* = 0.00018; Supporting Information Data S6). Moreover, across both sexes, there was a negative relationship between the internal chromatic contrast of the forewing and the concentration of cyanogenic glucosides (linear model, *F*_1,16_ = 29.77, *p* = 0.000053; Supporting Information Data S6).

### 3.2 Across species, there is no clear evidence of quantitative honesty

Despite a small number of species sampled, our phylogenetic tree (Figure 1) is in broad agreement with previously published phylogenies of the Zygaenidae and the genus *Zygaena* (Niehuis et al., 2006a, 2007). Using PGLS models to account for evolutionary relatedness, we found very few correlations between cyanogenic glucoside concentration and any of our measures of coloration (Supporting Information Data S5). Although trends followed the same direction whether males, females or all specimens were considered, the significance of these relationships did vary depending on sex (Table 3; Supporting Information Data S5). Moreover, significant correlations were not consistent between years (Table 3).

**Table 3:** Results of stepwise simplifications of PGLS models testing the relationship between cyanogenic glucoside concentration ([CNGlc]) and colour metrics, yielding a significant result with *λ* estimated by maximum likelihood (*λ *= 1 × 10^−6^), and re‐run with *λ *= 1 (Brownian motion model of evolution)

Data set	Model	Results with *λ *= 1 × 10^−6^	Results with *λ *= 1
2015, overall	[CNGlc] ~ luminance	*F*_1,7_ * *= 13.41, *p *= 0.0081	*F*_1,7_ * *= 5.45, *p *= 0.052
2015, males	[CNGlc] ~ luminance	*F*_1,6_ * *= 5.92, *p *= 0.051	*F*_1,6_ * *= 2.67, *p *= 0.15
2015, females	[CNGlc] ~ luminance	*F*_1,6_ * *= 14.98, *p *= 0.0083	*F*_1,6_ * *= 4.37, *p *= 0.082
2015, females	[CNGlc] ~ saturation	*F*_1,6_ * *= 11.78, *p *= 0.014	*F*_1,6_ * *= 3.56, *p *= 0.11
2015, females	[CNGlc] ~ hue	*F*_1,6_ * *= 15.68, *p *= 0.0075	*F*_1,6_ * *= 5.28, *p *= 0.061
2015, females	[CNGlc] ~ chromatic contrast	*F*_1,6_ * *= 13.71, *p *= 0.010	*F*_1,6_ * *= 4.58, *p *= 0.076
2016, overall	[CNGlc] ~ luminance contrast	*F*_1,9_ * *= 6.80, *p *= 0.028	*F*_1,9_ * *= 4.24, *p *= 0.070
2016, males	[CNGlc] ~ luminance contrast	*F*_1,8_ * *= 11.47, *p *= 0.0095	*F*_1,8_ * *= 11.61, *p *= 0.0093
2016, females	[CNGlc] ~ luminance contrast	*F*_1,6_ * *= 3.96, *p *= 0.094	*F*_1,6_ * *= 3.64, *p *= 0.11

**Figure 4:**
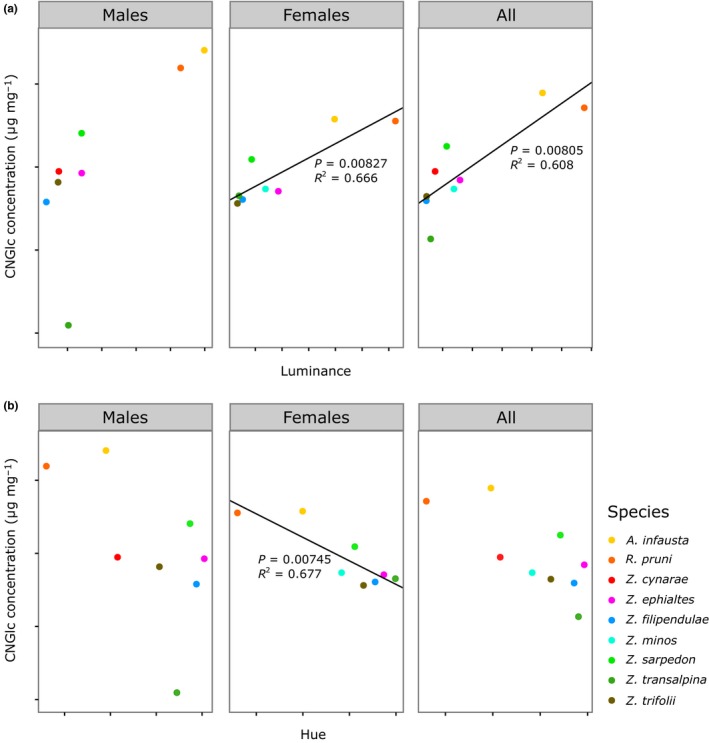
Mean cyanogenic glucoside (CNGlc) concentration and (a) luminance and (b) hue in species sampled in 2015, calculated in males, females and across both sexes. Lines represent the results of PGLS models

**Figure 5:**
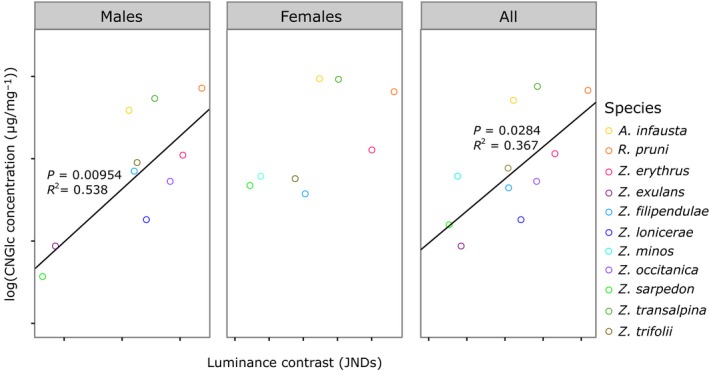
Mean log‐transformed cyanogenic glucoside (CNGlc) concentration and luminance contrast in species sampled in 2016, calculated in males, females and across both sexes. Lines represent the results of PGLS models

In addition, there were contrasting trends between luminance and colour, and most of the significant relationships between defences and certain signal properties were not indicative of quantitative honesty in the warning signals of these species. For samples collected in 2015, there was a positive correlation between luminance and cyanogenic glucoside concentration, suggesting that higher toxin levels were associated with paler markings (PGLS; across both sexes, *F*_1,7_ = 13.41, *p* = 0.0081; for females, *F*_1,6_ = 14.98, *p* = 0.0083; Figure 4a). This relationship was not significant for male samples, although the direction of the trend matched results in females and across both sexes (PGLS for males, *F*_1,7_ = 5.92, *p* = 0.051; Figure 4a). However, there was also a significant negative relationship, in females, between measures of colour (saturation, hue and chromatic contrast between markings and background colours) and cyanogenic glucoside levels (PGLS; saturation, *F*_1,6_ = 11.78, *p* = 0.014; hue, *F*_1,6_ = 15.68, *p* = 0.0075; chromatic contrast, *F*_1,6_ = 13.71, *p *= 0.010; Figure 4b), indicating that higher toxin levels correlated with less intense, potentially less red and less conspicuous markings, at least in terms of colour. In 2016, there was a positive correlation between internal luminance contrast and cyanogenic glucoside concentration, (PGLS; across both sexes, *F*_1,9_ = 6.80, *p* = 0.0029; in males, *F*_1,8_ = 11.47, *p* = 0.0095; Figure 5). This was not significant for females but the direction of the trend was consistent with those in males and across both sexes (*F*_1,6_ = 3.96, *p* = 0.094; Figure 5). The relationship between internal luminance contrast and the level of chemical defences could not be attributed to trends in marking luminance; unlike in 2015, there was no relationship between cyanogenic glucoside concentration and luminance, or any other colour metric in 2016 (Supporting Information Data S5).

Finally, maximum likelihood estimates found very little phylogenetic signal in the residuals of the regressions between colour metrics and cyanogenic glucoside levels (*λ* = 1 × 10^−6^ in each case). When *λ* was set to 1, corresponding to a Brownian model of evolution, only one relationship, the positive correlation between luminance contrast and cyanogenic glucoside levels in males in 2016, remained significant (*F*_1,8_ = 11.61, *p* = 0.0093; Table 3).

## 4 DISCUSSION

Overall, we found little evidence of quantitative signal honesty across the sampled species of Zygaenidae. Most colour metrics were not correlated with the concentration of defensive cyanogenic glucosides, whether male or female specimens were considered, and irrespective of the value of *λ* in phylogenetically controlled analyses (Table 3, Supporting Information Data S7). The trends that did emerge from this data set usually suggested a dishonest relationship between the strength of colour signals and defence levels, as higher toxin concentrations were associated with paler and less chromatically vibrant colours in 2015. Nevertheless, relationships between the concentration of cyanogenic glucosides and achromatic features could be seen to suggest quantitative honesty. When *λ* was estimated as a low value by maximum likelihood, some trends were significant in 2015, and, in particular, luminance was positively correlated with the concentration of cyanogenic glucosides across species. However, this did not lead to significant differences in achromatic contrast in the wings, and paler markings *per se* seem unlikely to constitute more salient markings. In terms of colour, only negative correlations with toxicity were found, suggesting dishonesty in signalling: saturation, hue and chromatic contrast were all negatively correlated with cyanogenic glucoside levels in females in 2015, especially in females. Under a Brownian motion model of evolution, we found only one significant relationship, a positive correlation in 2016 between luminance contrast and cyanogenic glucoside concentration across males of these species. This could be a potentially useful cue for predators, although there were no other significant correlations between other measures of coloration and toxin levels in that year.

### 4.1 Signal honesty across species – disentangling visual features

Assessing the relevance of these correlations to predator behaviour is difficult, as determining which aspects of signals and defences are most relevant to predators is not straightforward. Chemical defences are generally assessed by measuring toxin levels, but these may vary across body parts, total toxin amounts may be more relevant if prey are swallowed whole, and distastefulness, inducing taste rejection by predators (Skelhorn & Rowe, 2009, 2010) may not covary with toxicity: in nudibranchs, similarly distasteful red‐spotted species were shown to vary widely in their chemical profiles and lethality to brine shrimp (Winters et al., 2018). As cyanogenic glucosides are bitter‐tasting and can be dispensed to predators *via* defensive fluids during an attack (Jones, Rothschild, & Parsons, 1962), measuring levels of linamarin and lotaustralin in burnet moths should provide a relevant estimate of both unpalatability and toxicity. By contrast, the question of which properties of warning signals predators most attend to is still somewhat unresolved and is poorly studied in the context of the Zygaenidae.

Several lines of evidence suggest that chromatic features are the most important for avoidance learning, at least for avian predators (Stevens & Ruxton, 2012). In the laboratory, learning experiments, primarily with *Gallus gallus domesticus* chicks but also with *C. caeruleus* and other passerines, suggest that chromatic features are generally more important than pattern for avoidance learning, generalization and memory in birds (Aronsson & Gamberale‐Stille, 2008, 2012a; Exnerová et al., 2006; Kazemi, Gamberale‐Stille, Tullberg, & Leimar, 2014; Osorio, Jones, & Vorobyev, 1999; Osorio, Vorobyev, & Jones, 1999; Svádová et al., 2009). These findings are broadly supported by several artificial predation experiments in the wild, suggesting that colour is most critical in determining the survival of model prey exposed to avian predators, although pattern can have an added effect (Arenas et al., 2015; Finkbeiner, Briscoe, & Reed, 2014; Nokelainen, Hegna, Heudler, Lindstedt, & Mappes, 2012; Tan, Reid, & Elgar, 2016). As such, colour generally seems more important than luminance in predator avoidance, and several chromatic features are thought to be especially relevant to aposematic prey and their predators. Field studies with model frogs and ladybirds have shown that chromatic contrast to the natural background is particularly important (Arenas et al., 2015; Hegna, Saporito, Gerow, & Donnelly, 2011), while experiments presenting different species of Lycaeidae seed bug larvae to domestic chicks suggest that prey with redder and more saturated signals are more strongly avoided (Gamberale‐Stille & Tullberg, 1999). Long‐wavelength colours are also thought to be more effective as warning signals, due to innate avoidance by some predators and their greater stability under different lighting conditions (Arenas, Troscianko, & Stevens, 2014). Finally, experiments with artificial stimuli and natural prey items such as *Arctia plantaginis* (wood tiger moth) larvae suggest that larger coloured markings generate greater avoidance (Forsman & Merilaita, 1999; Lindstedt, Lindström, & Mappes, 2008; Lindström, Alatalo, Mappes, Riipi, & Vertainen, 1999; Smith, Halpin, & Rowe, 2014). In an honest signalling paradigm, we would thus expect stronger defences to be associated with stronger signals, represented by more saturated, redder, larger and more conspicuous markings (Arenas et al., 2015; Stevens & Ruxton, 2012). Yet, in our study, we found no association between marking size and toxicity across species, and the few correlations between chromatic features and toxicity we found in 2015 go against our expectations for quantitative honesty.

On the other hand, correlations between achromatic features, such as luminance and luminance contrast to wing background colours, could also be utilized by predators. Achromatic information may still be relevant to avian predators, potentially helping them to distinguish small pattern elements (Stevens, 2007), triggering initial avoidance of aposematic patterns (Sandre, Stevens, & Mappes, 2010) and speeding up learning (Aronsson & Gamberale‐Stille, 2012b). Luminance contrast in the pattern of prey items can also facilitate detection and avoidance learning in experiments with mantids (Prudic, Skemp, & Papaj, 2007), suggesting that it could be a useful cue for some invertebrate predators, to which burnet moths are also exposed (though note that mantids seem to lack colour discrimination, whereas many other invertebrates have good colour vision). In 2016, we found that internal luminance contrast was positively correlated with toxicity in males, so there is the potential for this signal property to act as an honest signal. Yet, it is also important to note that this trend was not linked to differences in marking luminance, so was likely to be driven by changes in the luminance of the dark background area of the moths’ wings. As the dark pigment melanin is involved in many other functions, from immune defences to thermoregulation (Solano, 2014), other selective pressures besides avoiding predation could be responsible for the trends in wing background luminance, and hence the relationship between luminance contrast and toxin levels. It would be useful to know more about the response of avian predators to the different features of a burnet moth‐like pattern, to conclusively determine whether any of the correlations found here could be relevant to predator behaviour in the wild. Across the board, comprehensively examining variation in many aspects of their colour signals suggests a lack of quantitative honesty across the zygaenid species studied here, but features such as luminance contrast between wing markings and background colours may be worthy of further investigation.

The above conclusions across species are broadly supported by results found when testing quantitative honesty within species in the Zygaenidae. In *Z. filipendulae*, few significant associations emerged between measures of coloration and cyanogenic glucoside levels, and the trends that were uncovered were more indicative of a negative relationship between signal strength and toxicity: within some populations, higher cyanogenic glucoside concentrations were associated with paler markings, whereas across populations, higher toxin levels were found in females with smaller and paler markings (Briolat, Zagrobelny, et al., 2018). Within *Z. ephialtes*, we found a negative correlation between toxin levels and internal chromatic contrast, similarly suggesting a negative correlation between signal salience and defence levels. As in *Z. filipendulae*, there is also a negative relationship between the relative size of the red markings and cyanogenic glucoside concentration, such that more toxic females have smaller markings. However, this relationship is reversed in males, raising the possibility that the area of red markings could act as an honest signal of toxicity in males. Aside from this potentially interesting difference between sexes, which may be related to the overall smaller size of males, there is little evidence of quantitative honesty within the Zygaenidae studied so far. As already discussed in the case of *Z. filipendulae* (Briolat, Zagrobelny, et al., 2018), the highly aversive nature of cyanogenic glucosides and fluctuations in individual toxin content over a moth's lifetime, depending on reproductive events, might limit the usefulness of quantitative honesty in burnet moths. More data would be required to test within‐species variation in a greater number of zygaenid species and determine whether this is a family‐wide pattern.

Relatively few studies have explored the relationship between coloration and the levels of chemical defences across species while accounting for phylogeny as we do here (but see Cortesi & Cheney, 2010; Santos & Cannatella, 2011; Summers & Clough, 2001), so the present study makes a rare contribution to the field. While some species have very small sample sizes (*N* = 1 or *N* = 2), these were still included in the analysis as increasing the number of species is key to greater reliability in phylogenetic analyses. The absence of signal honesty in the Zygaenidae is contrary to the results of other studies of signal honesty across species, in ladybirds (Arenas et al., 2015) and nudibranchs (Cortesi & Cheney, 2010), as well as some work in poison frogs (Santos & Cannatella, 2011; Summers & Clough, 2001; but see Darst et al., 2006). It demonstrates that quantitative signal honesty is not ubiquitous across families of aposematic species. Across species, a range of factors, including different habitat or microhabitat features (Endler, 1993), predator communities (Endler & Mappes, 2004; Nokelainen, Valkonen, Lindstedt, & Mappes, 2014) and life‐history traits (Longson & Joss, 2006), are likely to impose different fitness costs and benefits on the production of both signals and defences. If these costs and benefits do not change in parallel, honest signalling may not be expected (Speed & Ruxton, 2007). In the Zygaenidae, the economics of signals and defences are likely to differ between species, as they vary in their means of acquiring toxins, as well as in their behaviour. Sampling host plants from collection sites wherever possible, we measured the cyanogenic glucoside content of plant tissues the larvae were likely to feed on (Supporting Information Data S2) to address this issue. Although not comprehensive, our results suggest that, among our samples, only *Z. filipendulae* and *Z. occitanica* were feeding on plants with high levels of cyanogenic glucosides. *Z. trifolii*,* Z. cynarae*,* R. pruni* and in some cases *A. infausta* may also have been able to both sequester the cyanogenic glucosides linamarin and lotaustralin from their host plants as well as synthesize them themselves (Davis & Nahrstedt, 1986; Zagrobelny et al., 2014), whereas the other species appear to have relied entirely on de novo synthesis. Moreover, behavioural differences between the species in the *Zygaena* genus and the others will modulate their exposure to predators. The Procridinae behave more like cryptic species, flying rapidly and seeking to evade capture, whereas red‐spotted burnet moths are much more sluggish (Hofmann & Tremewan, 2017) and highly visible. Finally, although many of these species do co‐exist in the wild, our samples were collected from many different locations, so were not exposed to the same community of predators.

### 4.2 Considerations for cross‐species studies of signal honesty

Sex‐specific trends in quantitative honesty found for *Z. filipendulae* (Briolat, Burdfield‐Steel, et al., 2018; Briolat, Zagrobelny, et al., 2018) and *Z. ephialtes* suggest that differences between sexes should be considered in studies of signal honesty. The costs and benefits of aposematic signalling may vary between males and females of warningly coloured species, due to size dimorphism, trade‐offs related to sexual signalling, and variation in habitat use and behaviour, modulating their exposure to predators. In sexually dimorphic seven‐spot ladybirds (*Coccinella septempunctata*), an honest relationship between elytra carotenoids and coccinelline levels was only found in females, a result attributed to greater resource limitation or greater benefits of aposematic signalling in the larger sex (Blount et al., 2012). Burnet moths are similarly sexually dimorphic, with larger females (Naumann, Tarmann, & Tremewan, 1999), but other factors may also affect the economics of aposematic signalling: although both sexes are highly visible at rest, males are generally more active (Naumann et al., 1999), and there is some limited evidence that visual signals could play a role in sexual signalling, at close range (Friedrich & Friedrich‐Polo, 2005; Koshio, 2003; Zagatti & Renou, 1984), and at certain times of day (Hofmann & Kia‐Hofmann, 2010). Across species, trends were broadly similar between sexes in this study, but the significance of these relationships varied, suggesting that ignoring differences between sexes could mask interesting results. This is an important consideration, as existing studies of quantitative honesty across aposematic species and populations do not analyse males and females separately, even in taxa in which males and females are known to differ (e.g. in ladybirds; Arenas et al., 2015).

Our study also revealed considerable variation, in both coloration and toxicity, between individuals collected in two different years. These differences are unlikely to be caused by inconsistencies in our experimental procedures. While caterpillars were raised under natural conditions during collection trips, subsequent rearing conditions were kept as consistent as possible between specimens collected in 2015 and 2016. Moreover, differences in colour between years were found even among *Z. trifolii* specimens, collected as pupae from the same location and placed in an incubator with the same settings until eclosion, suggesting that conditions prior to euthanasia were not responsible for this variation. Preliminary experiments verified that the time that specimens were kept in the −80°C freezer between termination and photography did not impact coloration. Methods and equipment used for image capture did not vary between years, and all images from both seasons were processed and analysed together. Finally, we verified that differences in toxin levels were not caused by variations in the sensitivity of the LC‐MS machine and column used, by re‐running a subset of samples from both years together. While existing studies of signal honesty in aposematic species do not consider temporal variation in signal and defence traits, our study suggests that seasonal variation may have an impact on these traits.

With only 2 years of data, it is difficult to explain the observed patterns of between‐year variation, but environmental conditions, linked to variation in weather across years (see Supporting Information Data S7), are likely to impact investment in coloration and chemical defences in burnet moths. Variation in coloration in tiger moths (Erebidae) has been linked to fluctuations in local ecological conditions (Galarza, Nokelainen, Ashrafi, Hegna, & Mappes, 2014), and in particular temperature (Goulson & Owen, 1997; Lindstedt, Lindström, & Mappes, 2009). Climate may also indirectly affect resource allocation to signals and defences in aposematic species, via effects on their host plants. Cyanogenic plants possess highly variable levels of defensive chemicals, strongly affected by environmental conditions (Gleadow & Woodrow, 2002). The effects of temperature have been well documented in both *Trifolium repens* (white clover; Daday, 1954a,b, 1958; De Aráujo, 1976; Stochmal & Oleszek, 1997; Richards & Fletcher, 2002) and *Lotus corniculatus* (bird's foot trefoil), a key host plant of several Zygaenidae (Ellis, Keymer, & Jones, 1977; Jones, 1977; Salgado, Suchan, Pellissier, Rasmann, & Ducrest, 2016). For the species relying completely on de novo synthesis of cyanogenic glucosides, plant productivity may still be important. For example, nitrogen limitation will lead to reduced investment in cyanogenic glucosides in burnet moths, due to trade‐offs with other products, as suggested by the hypothesized breakdown of cyanogenic glucosides during pupation to fuel chitin synthesis (Zagrobelny et al., 2007). Interestingly, all the species in which cyanogenic glucoside levels decreased between years in males (*A. infausta*,* R. pruni* and *Z. sarpedon*) feed on acyanogenic host plants, suggesting that resource allocation trade‐offs may broadly differ between species able to sequester cyanogenic glucosides from their host plants and those who cannot. Comparing host plant levels of cyanogenic glucosides and other nutritional resources to moth toxin levels and coloration across years would help elucidate the relationship between environmental conditions, host properties and aposematic phenotypes. This type of longitudinal study could be a valuable means of testing for quantitative honesty in aposematic signalling, providing the opportunity to study how resources are allocated to signals and defences in response to environmental conditions, and as the communities of predators and prey co‐evolve.

In conclusion, the present work deepens our understanding of the relationship between signals and defences across species, by contributing to the small number of studies testing signal honesty across closely related aposematic species, with sophisticated methods for quantifying chemical defences, phylogenetic controls and measures of coloration accounting for predator vision. We find no clear evidence of quantitative signal honesty across the sampled species of Zygaenidae, especially not with regard to those aspects of appearance most likely to be salient to predators, a result likely attributable to varying costs of signal and defence production across species. Our study also highlights the importance of considering differences between sexes and temporal variation in analyses of signal honesty moving forward.

## CONFLICT OF INTEREST

All the authors of this work declare no conflict of interest.
