# Supplementary material for: No evidence of quantitative signal honesty across species of aposematic burnet moths (Lepidoptera: Zygaenidae)
Source: J Evol Biol. 2018 Nov 2;32(1):31–48. doi: 10.1111/jeb.13389 (PMC6378400; doi:10.1111/jeb.13389)
Supplement: Supplementary file 1 [file JEB-32-31-s001.docx]

**Supporting Information**

**Supporting Information S1: Details of locations for field collections.**

Table S1: Coordinates of field sites and number of specimens collected (N).

| Species | Year | Origin | Country | Latitude | Longitude | Altitude (m) | N |
| --- | --- | --- | --- | --- | --- | --- | --- |
| *A. infausta* | 2015 | Antigny | France | 47.0454 | 4.3524 | 420 | 3 |
|  | 2015 | La Faurie | France | 44.3401 | 5.4419 | 820 | 2 |
|  | 2015 | Le Fournas | France | 44.0753 | 5.9722 | 480 | 5 |
|  | 2015 | Nolay | France | 46.9293 | 4.6656 | 455 | 4 |
|  | 2015 | Sully | France | 47.1738 | 4.3748 | 490 | 7 |
|  | 2016 | Antigny | France | 47.0454 | 4.3524 | 420 | 15 |
|  | 2016 | Andon | France | 43.4534 | 6.5140 | 1110 | 2 |
| *R. pruni* | 2015 | Antigny | France | 47.0454 | 4.3524 | 420 | 7 |
|  | 2015 | Lardier-et-Valenca | France | 44.2512 | 5.5689 | 830 | 1 |
|  | 2016 | Lusigny-sur-Ouche | France | 47.0831 | 4.6744 | 400 | 2 |
|  | 2016 | Nolay | France | 46.9293 | 4.6656 | 455 | 6 |
| *T. ampellophaga* | 2016 | Biot* | France | 43.6269 | 7.0981 | 60 | 1 |
| *Z. cynarae* | 2015 | Mouans-Sartoux* | France | 43.6204 | 6.9725 | 150 | 1 |
| *Z. ephialtes* | 2015 | Corzé | France | 47.5395 | -0.3413 | 50 | 21 |
| *Z. erythrus* | 2016 | Garriguet-Ste- Eulalie | France | 43.9866 | 4.3052 | 90 | 8 |
|  | 2016 | Le Fournas | France | 44.0753 | 5.9722 | 480 | 3 |
| *Z. exulans* | 2016 | Molines-en-Queyras | France | 44.7018 | 6.8208 | 2600 | 5 |
| *Z. filipendulae* | 2015 | Cabasse | France | 43.4201 | 6.2355 | 250 | 4 |
|  | 2015 | Holywell Bay | UK | 50.3910 | -5.1430 | 20 | 23 |
|  | 2015 | Lamorna Cove | UK | 50.0610 | -5.5544 | 30 | 25 |
|  | 2015 | Lardier-et-Valenca | France | 44.2512 | 5.5689 | 830 | 5 |
|  | 2015 | Le Fournas | France | 44.0753 | 5.9722 | 480 | 1 |
|  | 2015 | Mouans-Sartoux* | France | 43.6204 | 6.9725 | 150 |  |
|  | 2015 | Pendeen Watch | UK | 50.1636 | -5.6705 | 60 | 9 |
|  | 2015 | Porthnanven | UK | 50.1157 | -5.6996 | 20 | 5 |
|  | 2015 | St Félix de Tournegat | France | 43.1312 | 1.7483 | 310 | 2 |
|  | 2015 | Taastrup | Denmark | 55.6346 | 12.2625 | 30 | 25 |
|  | 2015 | Upton Towans | UK | 50.2100 | -5.3972 | 40 | 2 |
|  | 2015 | Veynes | France | 44.3239 | 5.4924 | 900 | 4 |
|  | 2016 | La Chapelle-en-Valgaudémar | France | 44.8169 | 6.1953 | 2100 | 1 |
|  | 2016 | Les Piles, La Saulce | France | 44.4432 | 6.0288 | 600 | 1 |
|  | 2016 | Mortiès* | France | 43.7700 | 3.8223 | 200 | 1 |
|  | 2016 | St Bauzille* | France | 43.3542 | 3.3233 | 140 |  |
|  | 2016 | St-Cézaire-sur-Siagne | France | 43.3848 | 6.4928 | 450 |  |
|  | 2016 | Vacquières* | France | 43.8445 | 3.9436 | 110 | 2 |
| *Z.lonicerae* | 2016 | Roubion | France | 44.0529 | 7.0301 | 1270 | 1 |
| *Z. minos* | 2015 | Le Cialancier | France | 44.2091 | 6.9781 | 1000 | 1 |
|  | 2016 | Le Cialancier | France | 44.2091 | 6.9781 | 1000 | 1 |
| *Z.occitanica* | 2016 | Mouans-Sartoux* | France | 43.6204 | 6.9725 | 150 | 1 |
|  | 2016 | Callian | France | 43.3829 | 6.4630 | 430 | 1 |
| *Z.sarpedon* | 2015 | La Faurie | France | 44.3401 | 5.4419 | 820 | 6 |
|  | 2016 | Le Fournas | France | 44.0753 | 5.9722 | 480 | 2 |
| Species | **Year** | **Origin** | **Country** | **Latitude** | **Longitude** | **Altitude (m)** | **N** |
| *Z. transalpina* | 2015 | Mazaugues* | France | 43.3486 | 5.9225 | 690 | 1 |
|  | 2015 | Mouans-Sartoux* | France | 43.6204 | 6.9725 | 150 | 2 |
| *Z. transalpina* | 2016 | Mazaugues* | France | 43.3486 | 5.9225 | 690 | 12 |
|  | 2016 | Roubion | France | 44.0529 | 7.0301 | 1270 | 1 |
| *Z. trifolii* | 2015 | Bostraze Bog | UK | 50.1304 | -5.6501 | 160 | 7 |
|  | 2015 | Loggans Moor | UK | 50.2021 | -5.3979 | 20 | 2 |
|  | 2016 | Bostraze Bog | UK | 50.1304 | -5.6501 | 160 | 14 |

*: location to nearest town/village


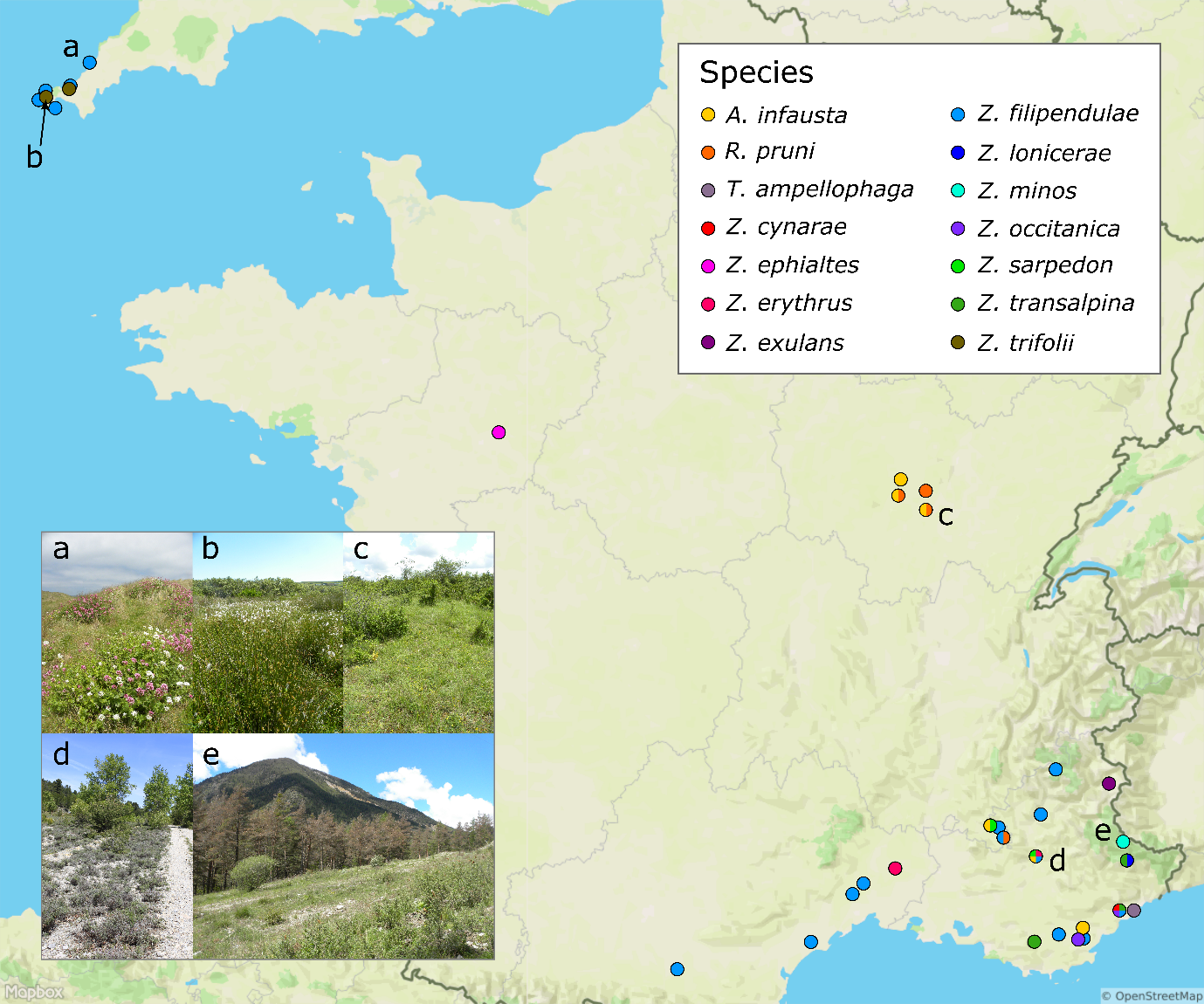


Figure S1: Map of collection localities colour-coded by species, illustrating five example habitats: (a) Holywell Bay, UK, (b) Bostraze Bog, UK, (c) Antigny, France, (d) Le Fournas, France, (e) Le Cialancier, France. The Danish locality where *Z. filipendulae* was collected is not represented. The colour of each circle represents the species collected in that locality (see key); circles with multiple colours indicate that several different species were found there.

**Supporting Information S2: Cyanogenic glucosides in Zygaenidae host plants**

Table S2: Concentration of linamarin and lotaustralin in host plants sampled during larval field collections. Plant samples were weighed then preserved in 1ml 80% MeOH prior to LC-MS, following the same protocol as for moth samples.

| **Plant species** | **Mean [linamarin] (μg/mg) ± s.d.** | **Mean [lotaustralin] (μg/mg) ± s.d.** | **Sample number** | **Plant provenance** | **Zygaenidae hosts** |
| --- | --- | --- | --- | --- | --- |
| *Cotoneaster* sp. | 0 | 0 | 3 | Commercial (Truffaut, France) | *A. infausta* |
| *Crataegus* sp. | 0 | 0 | 3 | Penryn, Cornwall, UK | *A. infausta* |
| *Dorycnium pentaphyllum* | 2.809*10^-1^  ± 6.783*10^-2^ | 2.958  ± 1.357 | 3 | Commercial (Pic Vert nurseries, France) | *Z. filipendulae, Z. occitanica* |
| *Eryngium campestre* | 0 | 0 | 3 | Fondurane, France | *Z. erythrus, Z. sarpedon* |
| *Hippocrepis comosa* | 0 | 0 | 3 | Fondurane, France | *Z. filipendulae, Z. transalpina* |
| *Lotus corniculatus* | 4.815*10^-1^  ± 5.999*10^-1^ | 1.792  ± 2.555 | 32 (3-5 per location) | Commercial (Wildflower Shop, UK);Holywell Bay, Lamorna Cove, Pendeen Watch, Porthnanven, Upton Towans (all Cornwall, UK); Taastrup, Denmark; Nolay, Vauchinon, Souvert (all France) | *Z. filipendulae* |
| *Lotus pedunculatus* | 8.797*10^-3^  ± 3.458*10^-3^ | 7.125*10^-3^  ± 2.801*10^-3^ | 6 (3 per location) | Bostraze Bog; Loggans Moor (Cornwall, UK) | *Z. trifolii* |
| *Peucedanum cervaria* | 3.250*10^-3^  ± 2.801*10^-3^ | 1.236*10^-2^  ± 9.650*10^-3^ | 3 | Mouans-Sartoux, France | *Z. cynarae* |
| *Pimpinella saxifraga* | 1.834*10^-4^  ± 3.177*10^-4^ | 0 | 3 | Commercial (Amazon, UK) | *Z. minos* |
| *Prunus* sp. | 1.238*10^-2^  ± 1.606*10^-2^ | 4.944*10^-2^  ± 6.620*10^-2^ | 6 (3 per location) | Antigny; Nolay (Bourgogne, France) | *A. infausta, R. pruni* |
| *Securigera varia* | 0 | 0 | 6 (3 per location) | Corzé; Roubion (France) | *Z. ephialtes, Z. transalpina* |
| *Trifolium* sp. | 0 | 0 | 3 | Le Cialancier, France | *Z. lonicerae* |
| *Vitis* sp. | 0 | 0 | 3 | Mougins, France | *T. ampellophaga* |

**Supporting Information S3: Detailed methods for image analysis and calculation of colour metrics**

*Image analysis*

All image analysis was performed in ImageJ (Schneider *et al*., 2012) using open access custom-made plugins in the Image Calibration and Analysis Toolbox (Troscianko & Stevens, 2015). To account for nonlinearity inherent in the camera set-up and fluctuations in lighting conditions, the images were linearised and normalised, thereby ensuring accurate and objective measures of coloration (Stevens *et al*., 2007). The photographs were also scaled to 100 pixels/mm, to enable comparative measurements of the size of coloured areas across specimens. Photographs taken with the two types of filter were combined using the automatic alignment tool in the Image Calibration and Analysis Toolbox (Troscianko & Stevens, 2015), ultimately yielding a multispectral image for each specimen. This consists in a stack of 32-bit image slices, each representing reflectance values in the different channels of camera vision: the red (R), green (G) and blue (B) channels in the visible spectrum, and red (R) and blue (B) channels for ultraviolet wavelengths (see Troscianko & Stevens, 2015).

Anecdotal observations suggest that several bird species will attack adult Zygaenidae in the wild (Tremewan, 2006; Morelli, 2013). These include *Cuculus canorus* (common cuckoos), and the passerines *S. vulgaris*, *A. arvensis*, *A. pratensis*, *Turdus merula* (blackbirds), *Passer domesticus* (house sparrows), *Emberiza* spp. (buntings), *Saxicola rubetra* (whinchats), *Lanius collurio* (red-backed shrikes) and *Pyrrhocorax* spp. (choughs). To quantify moth coloration from the perspective of their most likely predators, the wing photographs were therefore converted to a typical form of avian vision. Using the spectral sensitivities of *Cyanistes caeruleus* (blue tit), the model species for the ultraviolet-sensitive (UVS) passerine visual system (Hart *et al*., 2000) and a polynomial mapping technique, the image layers for each specimen were transformed to the predicted cone catch values for each of the five avian cone types: long wavelength- (LW-), medium wavelength- (MW-), short wavelength- (SW-) and ultraviolet- (UV-) sensitive photoreceptors, and double cones. This method for converting camera values to cone catches has previously been validated with a database of reflectance spectra from natural stimuli (Arnold *et al*., 2010), and shown to achieve highly accurate matches compared to approaches using reflectance spectra (Stevens & Cuthill, 2006; Pike, 2011; Troscianko & Stevens, 2015).

All the red markings and dark background areas of the forewings were selected for analysis using the freehand tool in Image J. The whole forewing was also selected, to enable us to calculate the percentage of wing area occupied by the wing markings. Two of the species collected do not display red forewing markings: for *Rhagades pruni* (Denis & Schiffermüller, 1775) individuals, the iridescent blue patch at the base of the wing was selected as the markings, while for *Theresimima ampellophaga* (Bayle-Barelle, 1808) the whole uniform wing was measured as a single patch. Cone catch values for every photoreceptor type were obtained from each selected patch, then averaged to obtain a single measure of colour per individual, for both the wing markings and wing background area.

*Colour metrics*

Based on the average cone catch values, several measures of coloration were calculated: luminance, saturation, and hue of the forewing marking colours, as well as both chromatic and luminance contrasts between markings and background colours. Luminance provides a measure of perceived lightness and is equal to the cone catch value for the double cones, a type of photoreceptor considered responsible for the detection of achromatic cues in birds (Jones & Osorio, 2004; Osorio & Vorobyev, 2005). Saturation, a measure of colour intensity or richness (e.g. on a scale from pink to red) was obtained by plotting the wing colours in a tetrahedral avian colour space, with XYZ coordinates derived from the standardised cone catch values to the LW-, MW-, SW- and UV-sensitive photoreceptors (following equations in Endler & Mielke, 2005). Saturation is then calculated as the Euclidian distance between the colour of interest and the centre of the tetrahedron, as per methods used in many studies of animal coloration (e.g. Stoddard & Prum, 2008; Stevens *et al*., 2014a,b; Arenas & Stevens, 2017). Hue provides a sense of the type of a colour (e.g. red or blue), and was calculated as a ratio of standardised cone catch values to the four colour channels (LW, MW, SW and UV), an approach that broadly reflects the importance of colour opponent channels for visual processing in many animals, including birds (Osorio *et al*., 1999). While the exact opponent channels used in avian vision are not known, principal component analysis (PCA) can be used to determine the main axes of colour variation among sampled colours and inform intuitive colour channels to calculate hue (Spottiswoode & Stevens, 2011; Stevens *et al*., 2014a,b). PCA was performed on a covariance matrix of the standardised cone catches of all colour patches measured, for the LW, MW, SW and UV channels (after Spottiswoode & Stevens, 2011). The first principal component accounted for over 91% of variation in marking colour, and consequently was used to derive a single ratio of cone catch values, with the following equation: Hue = (LW+UV)/(SW+MW). While this ratio is inspired by the concept of colour opponency, it provides only a sense of variation in coloration in the samples measured here, and may not reflect actual opponent channels in the avian visual system. In this dataset, higher hue values correspond to colours with relatively greater reflectance in the LW and/or UV colour channel than in the SW and MW channels, so indicate redder colours, higher ultraviolet reflectance, or both.

Internal contrasts between the wing ground colour and wing marking colour were calculated using a log version of the Vorobyev-Osorio model (Vorobyev & Osorio, 1998). This model takes into account the sensitivity and abundance of each photoreceptor type for the relevant visual system, as well as noise between the photoreceptors, to calculate a measure of how easily two colours are likely to be discriminated. For chromatic contrast, we used relative cone abundance values for *Cyanistes caeruleus* as a model for the UVS avian visual system (UV=1, SW=1.92, MW=2.68, LW=2.7; Hart *et al.*, 2000), and a widely-used estimate of the Weber fraction (ω=0.05; Eaton, 2005; Håstad *et al*., 2005; Stevens, 2011) to calculate noise. Achromatic, or luminance, contrast was taken as the natural logarithm of the ratio between the mean double cone catch values of two colours, divided by the same Weber fraction (Siddiqi *et al*., 2004). For both types of contrast, these calculations yield a value for discriminability, measured in “just-noticeable differences” (JNDs), whereby pairs of colours with values below a threshold of 1 are indiscriminable, even in optimal lighting conditions, while colours with values greater than 1 are increasingly easy to tell apart, in worsening lighting conditions (Siddiqi *et al*., 2004).

**Supporting Information S4: Details for sequences used in phylogenetic reconstruction.**

Table S4: EMBL accession numbers for the sequences used in this study.

| **Taxon** | **Accession numbers** | | | | |
| --- | --- | --- | --- | --- | --- |
|  | ND1 | 16S rRNA^*^ | 12S rRNA | 18S rRNA | 28S rRNA |
| Sesiidae  *Sesia bembeciformis* (Hübner,1806) | AJ844306 | AJ831588 | AJ785615 | AJ830746 | AJ844024 |
| Zygaenidae – Chalcosiinae  *Aglaope infausta* (Linnaeus, 1767) | AJ844314 | AJ831596 | AJ785623 | AJ830754 | AJ844032 |
| Zygaenidae – Procridinae  *Rhagades pruni* (Denis & Schiffermüller, 1775) | AJ844324 | AJ831606 | AJ785633 | AJ830764 | AJ844042 |
| *Theresimima ampellophaga*  (Bayle-Barelle, 1808) | AJ844325 | AJ831607-8 | AJ785634 | AJ830765 | AJ844043 |
| Zygaenidae – Zygaeninae  *Zygaena cynarae samarensis* (Holik, 1939) | AJ844389 | AJ831677 | AJ785698 | AJ830829 | AJ844107 |
| *Zygaena ephialtes albaflavens* (Verity, 1920) | AJ844427 | AJ831722 | AJ785736 | AJ830867 | AJ844145 |
| *Zygaena erythrus actae* (Burgeff, 1926) | AJ844390 | AJ831678 | AJ785699 | AJ830830 | AJ844108 |
| *Zygaena exulans exulans* (Hohenwarth, 1792) | AJ844428 | AJ831723 | AJ785737 | AJ830868 | AJ844146 |
| *Zygaena filipendulae gemina* (Burgeff, 1914) | AJ844429 | AJ831724 | AJ785738 | AJ830869 | AJ844147 |
| *Zygaena lonicerae leonensis* (Tremewan, 1961) | AJ844433 | AJ831728 | AJ785742 | AJ830873 | AJ844151 |
| *Zygaena minos ingens* (Burgeff, 1926) | AJ844407 | AJ831698 | AJ785716 | AJ830847 | AJ844125 |
| *Zygaena occitanica huescacola*  (Tremewan & Manley, 1965) | AJ844362 | AJ831649 | AJ785671 | AJ830802 | AJ844080 |
| *Zygaena sarpedon lusitanica* (Reiss, 1936) | AJ844418 | AJ831713 | AJ785727 | AJ830858 | AJ844136 |
| *Zygaena transalpina hippocrepidis*  (Hübner, 1799) | AJ844442 | AJ831737 | AJ785751 | AJ830882 | AJ844160 |
| *Zygaena trifolii diffusemarginata*  (Rothschild, 1933) | AJ844444 | AJ831739 | AJ785753 | AJ830884 | AJ844162 |

*: Including tRNA-Leu and tRNA-Val

**Supporting Information S5: Results of Phylogenetic Generalised Least Squares (PGLS) models testing the relationship between cyanogenic glucoside concentration and coloration in Zygaenidae, for each combination of collection years and sex (Tables a-f), with λ estimated by maximum likelihood.** Full models were simplified by stepwise simplification with a significance threshold level α=0.05. Significant relationships are highlighted in italics. ILC=Internal Luminance Contrast, ICC=Internal Chromatic Contrast. In all cases, maximum likelihood found λ=1*10^-6^.

Table S5a: Samples collected in 2015, males

| **Variables included in full models** | Luminance, Marking size, ILC, ICC | | Luminance, Marking size, ILC, Saturation | | Luminance, Marking size, ILC, Hue | |
| --- | --- | --- | --- | --- | --- | --- |
| **Model simplification** | ILC | F_1,3_=0.0007, p=0.98 | ILC | F_1,3_=0.0021, p=0.97 | ILC | F_1,3_=0.054, p=0.81 |
|  | ICC | F_1,4_=1.17, p=0.34 | Saturation | F_1,4_=1.74, p=0.26 | Hue | F_1,4_=0.47, p=0.53 |
|  | Marking size (%) | F_1,5_=3.88, p=0.11 | Marking size (%) | F_1,5_=3.88, p=0.11 | Marking size (%) | F_1,5_=3.88, p=0.11 |
|  | Luminance | F_1,6_=5.92, p=0.051 | Luminance | F_1,6_=5.92, p=0.051 | Luminance | F_1,6_=5.92, p=0.051 |

Table S5b: Samples collected in 2015, females

| **Variables included in full models** | Marking size, ILC, Luminance | | Marking size, ILC, Hue | | Marking size, ILC, Saturation | | Marking size, ILC, ICC | |
| --- | --- | --- | --- | --- | --- | --- | --- | --- |
| **Model simplification** | ILC | F_1,4_=0.73, p=0.44 | ILC | F_1,4_=0.11, p=0.76 | ILC | F_1,4_=0.0031, p=0.96 | ILC | F_1,4_=0.058, p=0.82 |
|  | Marking size (%) | F_1,5_=1.22, p=0.32 | Marking size (%) | F_1,5_=0.75, p=0.43 | Marking size (%) | F_1,5_=0.13, p=0.73 | Marking size (%) | F_1,5_=0.18, p=0.69 |
|  | Luminance | *F_1,6_=14.98 p=0.0083* | Hue | *F_1,6_=15.68, p=0.0075* | Saturation | *F_1,6_=11.78, p=0.014* | ICC | *F_1,6_=13.71, p=0.010* |

Table S5c: Samples collected in 2015, both sexes combined

| **Variables included in full models** | Luminance, Marking size, ILC, Hue | | Luminance, Marking size, ILC, ICC | | Luminance, Marking size, ILC, Saturation | |
| --- | --- | --- | --- | --- | --- | --- |
| **Model simplification** | Hue | F_1,4_=0.038, p=0.86 | ICC | F_1,4_=0.025, p=0.88 | Saturation | F_1,4_=0.0001, p=0.99 |
|  | Marking size (%) | F_1,5_=0.82, p=0.41 | Marking size (%) | F_1,5_=0.82, p=0.41 | Marking size (%) | F_1,5_=0.82, p=0.41 |
|  | ILC | F_1,6_=4.35, p=0.082 | ILC | F_1,6_=4.35, p=0.082 | ILC | F_1,6_=4.35, p=0.082 |
|  | Luminance | *F_1,7_=13.41, p=0.0081* | Luminance | *F_1,7_=13.41, p=0.0081* | Luminance | *F_1,7_=13.41, p=0.0081* |

Table S5d: Samples collected in 2016, males

| **Variables included in full models** | Luminance, Marking size, ILC, Hue | | Luminance, Marking size, ILC, Saturation | | Luminance, Marking size, ILC, ICC | |
| --- | --- | --- | --- | --- | --- | --- |
| **Model simplification** | Luminance | F_1,5_=0.0009, p=0.98 | Marking size (%) | F_1,5_=0.047, p=0.84 | Luminance | F_1,5_=0.14, p=0.73 |
|  | Marking size (%) | F_1,6_=0.28, p=0.61 | Saturation | F_1,6_=0.24, p=0.64 | Marking size (%) | F_1,6_=0.31, p=0.60 |
|  | Hue | F_1,7_=1.18, p=0.31 | Luminance | F_1,7_=1.19, p=0.31 | ICC | F_1,7_=1.66, p=0.24 |
|  | ILC | *F_1,8_=11.47, p=0.0095* | ILC | *F_1,8_=11.47, p=0.0095* | ILC | *F_1,8_=11.47, p=0.0095* |

Table S5e: Samples collected in 2016, females

| **Variables included in full models** | Luminance, Marking size, ILC, Hue | | Luminance, Marking size, ILC, Saturation | | Luminance, Marking size, ILC, ICC | |
| --- | --- | --- | --- | --- | --- | --- |
| **Model simplification** | Luminance | F_1,3_=0.095, p=0.78 | Luminance | F_1,3_=0.021, p=0.89 | Luminance | F_1,3_=0.15, p=0.72 |
|  | Hue | F_1,4_=0.039, p=0.85 | Saturation | F_1,4_=0.057, p=0.82 | ICC | F_1,4_=0.057, p=0.82 |
|  | Marking size (%) | F_1,5_=1.14, p=0.33 | Marking size (%) | F_1,5_=1.14, p=0.33 | Marking size (%) | F_1,5_=1.14, p=0.33 |
|  | ILC | F_1,6_=3.96, p=0.094 | ILC | F_1,6_=3.96, p=0.094 | ILC | F_1,6_=3.96, p=0.094 |

Table S5f: Samples collected in 2016, both sexes combined

| **Variables included in full models** | Luminance, Marking size, ILC, Hue | | Luminance, Marking size, ILC, ICC | | Luminance, Marking size, ILC, Saturation | |
| --- | --- | --- | --- | --- | --- | --- |
| **Model simplification** | Hue | F_1,6_=0.0001, p=0.99 | Luminance | F_1,6_=0.042, p=0.85 | Saturation | F_1,6_=0.32, p=0.59 |
|  | Marking size (%) | F_1,7_=0.26, p=0.63 | Marking size (%) | F_1,7_=0.087, p=0.78 | Marking size (%) | F_1,7_=0.26, p=0.63 |
|  | Luminance | F_1,8_=0.72, p=0.42 | ICC | F_1,8_=1.03, p=0.34 | Luminance | F_1,8_=0.72, p=0.42 |
|  | ILC | *F_1,9_=6.80, p=0.028* | ILC | *F_1,9_=6.80, p=0.028* | ILC | *F_1,9_=6.80, p=0.028* |

**Supporting Information S6: Intra-specific variation – a case-study in *Z. ephialtes***

Table S6: Results of stepwise simplification of a linear model testing the relationship between cyanogenic glucoside levels and multiple colour metrics in *Z. ephialtes*. Only the model including saturation is presented, as replacing saturation by hue in the full model yielded the same conclusions. Significance levels: *:p<0.05, **:p<0.01, ***:p<0.001.

| **Factor** | **F** | **df** | **P** | **Significance** |
| --- | --- | --- | --- | --- |
| Luminance contrast : Sex | 0.070 | 1,9 | 0.80 | n.s. |
| Luminance : Sex | 2.73 | 1,10 | 0.13 | n.s. |
| Saturation : Sex | 3.92 | 1,11 | 0.073 | n.s. |
| Chromatic contrast : Sex | 0.82 | 1,12 | 0.38 | n.s. |
| Luminance contrast | 0.46 | 1,13 | 0.51 | n.s. |
| Luminance | 0.33 | 1,14 | 0.57 | n.s. |
| Saturation | 1.25 | 1,15 | 0.28 | n.s. |
| Relative marking size : Sex | 23.50 | 1,16 | *0.00018* | *** |
| Chromatic contrast | 29.77 | 1,16 | *0.000053* | *** |


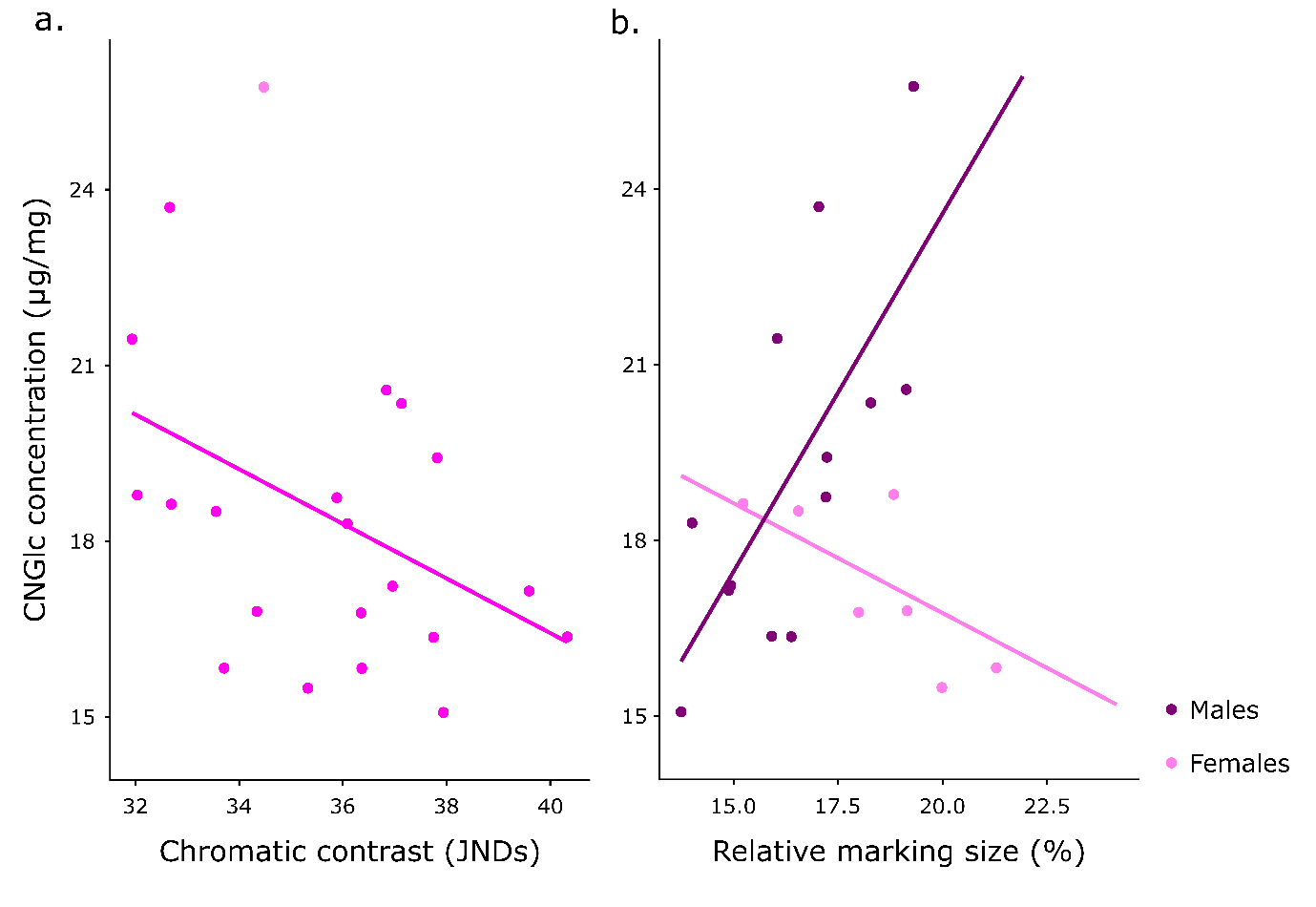


Figure S6: Relationship between cyanogenic glucoside levels and (a) chromatic contrast and (b) relative marking size in *Z. ephialtes* samples.

**Supporting Information S7: Climate data for collection sites in 2015 and 2016**

*Methods*

Climate data (monthly mean, minimum, and maximal temperature, total rainfall and total hours of sunshine in 2015 and 2016) were collated from publicly-available historical observations databases from the Met Office and Météo France. Eight weather stations, closest to all field collection sites in the UK and France, were selected for analysis (Table S7). Data from the three coldest months of the year (December, January, and February) and the three months prior to specimen collection (March, April, and May) were examined to provide a sense of winter and growing season conditions respectively. Each climate variable was analysed using a linear mixed effects model (LME), including year as a fixed effect and month and location as random effects, using the package ‘lme4’ (Bates *et al.*, 2014). Model assumptions were checked with the mcp.fnc function in the ‘LMERConvenienceFunctions’ package (Tremblay and Ransijn, 2014).

Table S7: Locations of selected weather stations

| **Station name** | **Country (Region)** | **Latitude** | **Longitude** | **Altitude (m)** |
| --- | --- | --- | --- | --- |
| Camborne | United Kingdom (Cornwall) | 50.218 | -5.327 | 87 |
| Cap Cépet – 7661^*^ | France (Provence Alpes Côte d’Azur) | 43.079 | 5.941 | 115 |
| Dijon – 7280 | France (Bourgogne Franche-Comté) | 47.268 | 5.088 | 219 |
| Embrun – 7591 | France (Provence Alpes Côte d’Azur) | 44.566 | 6.502 | 871 |
| Montpellier – 7643 | France (Languedoc-Roussillon Midi-Pyrenées) | 43.577 | 3.963 | 2 |
| Nice – 7690 | France (Provence Alpes Côte d’Azur) | 43.649 | 7.209 | 2 |
| St Girons – 7627 | France (Languedoc-Roussillon Midi-Pyrenées) | 43.005 | 1.107 | 414 |
| Tours – 7240 | France (Centre – Val de Loire) | 47.445 | 0.727 | 108 |

^*^: Records of hours of sunshine were not available for this station.

*Results*

Even though the collection sites were widely distributed across France and in Cornwall (UK), in a range of habitat types, there were broad climatic differences between the two years across all localities. The winter preceding the first set of field collections (December 2014 – February 2015) was significantly colder than the second (December 2015 – February 2016), with lower mean minimum, mean, and maximum temperatures (LME, minimum temperature, (χ^2^)_1_=38.71, p=4.91*10^-10^; mean temperature, (χ^2^)_1_=42.64, p=6.59*10^-11^; maximum temperature, (χ^2^)_1_=24.92, p=5.98*10^-7^; Figure S7a). By contrast, spring 2015 was warmer, sunnier and drier than spring 2016 (LME, minimum temperature, (χ^2^)_1_=15.31, p=9.11*10^-5^; mean temperature, (χ^2^)_1_=22.47, p=2.13*10^-6^; maximum temperature, (χ^2^)_1_=19.44, p=1.04*10^-5^; hours of sun, (χ^2^)_1_=8.314, p=3.93*10^-3^; rainfall, (χ^2^)_1_=6.66, p=9.85*10^-3^; Figure S7b).


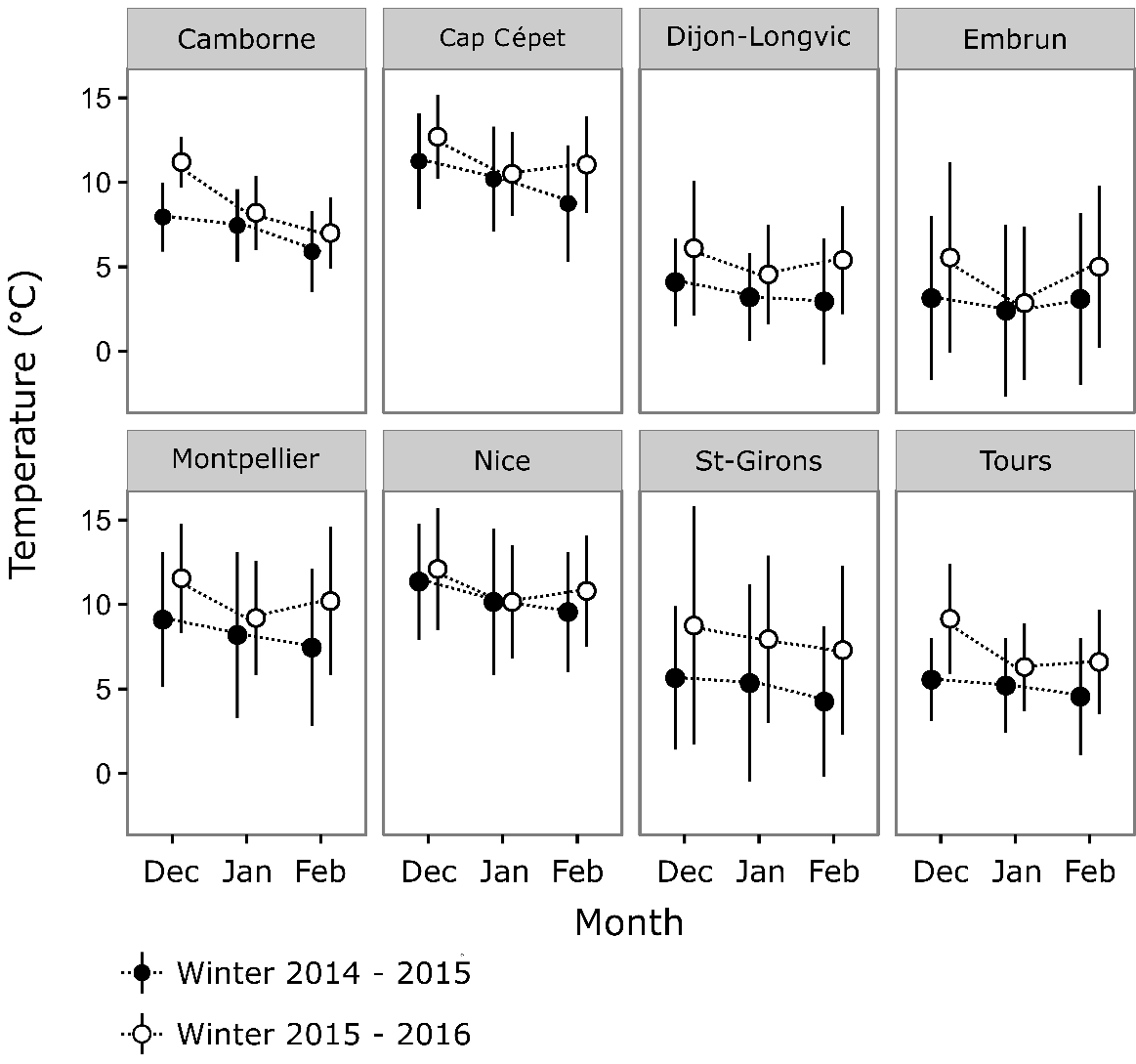


Figure S7a: Temperature ranges in the winters preceding the 2015 and 2016 collections. Circles indicate monthly mean temperature and solid lines represent mean minimum and maximum temperatures.


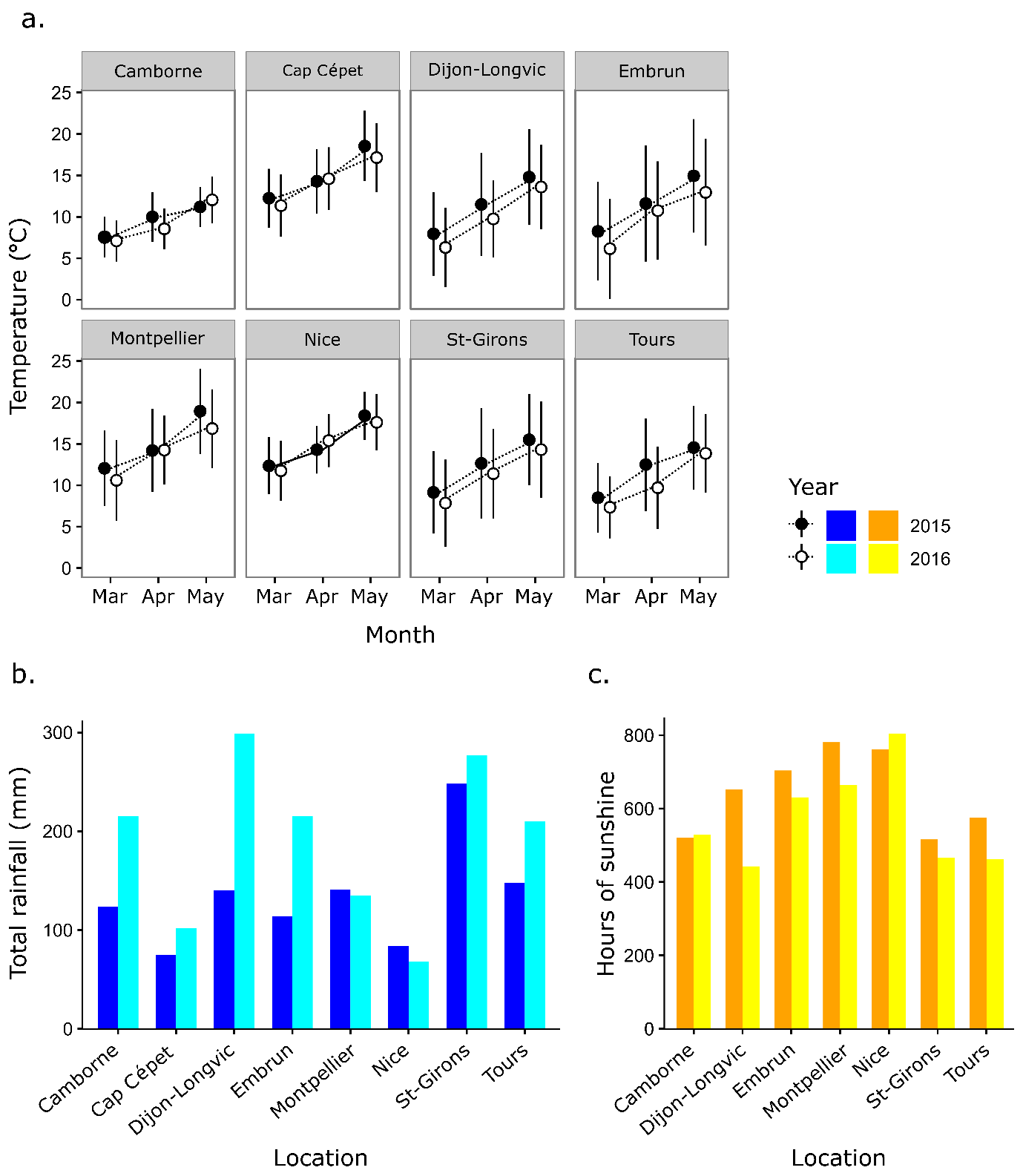


Figure S7b: Weather conditions in the growing season in 2015 and 2016. In (a), circles indicate monthly mean temperature and solid lines represent mean minimum and maximum temperatures.

*References*

Arnold, S.E., Faruq, S., Savolainen, V., McOwan, P.W. & Chittka, L. 2010. FReD: the floral reflectance database — a web portal for analyses of flower colour. *PLoS ONE* 5: e14287.

Bates, D., Maechler, M., Bolker, B. and Walker, S. 2014. lme4: Linear mixed-effects models using Eigen and S4. R package version 1.1-7. See <http://cran.r-project.org/package=lme4>.

Stevens, M., Lown, A.E. & Wood, L.E. 2014a. Color change and camouflage in juvenile shore crabs *Carcinus maenas*. *Front. Ecol. Evol.* 2: 1–14.

Stevens, M., Lown, A.E. & Wood, L.E. 2014b. Camouflage and individual variation in shore crabs (*Carcinus maenas*) from different habitats. *PLoS ONE* 9(12): e115586.

Tremblay, A. and Ransijn, J. 2014. lmerConveniencefunctions: a suite of functions to back-fit fixed effects and forward-fit random effects, as well as other miscellaneous functions. R package version 2.5. See <http://cran.r-project.org/web/packages/LMERConvenienceFunctions/index.html>
